# Dupilumab attenuate IL-4 and IL-13 synergistic effect on airway remodeling in asthma via blocking IL-4Rα/STAT6 axis

**DOI:** 10.1038/s41598-026-54625-7

**Published:** 2026-05-30

**Authors:** Lina Sahnoon, Rola Abujabal, Tasneem M. Alanta, Bushra Mdkhana, Ronald Olivenstein, Ellen Puré, Bassam Mahboub, Yves Laumonnier, Rifat Hamoudi, Khuloud Bajbouj, Qutayba Hamid

**Affiliations:** 1 Research Institute of Medical and Health Sciences, University of Sharjah, P.O. Box 27272, Sharjah, United Arab Emirates; 2 College of Medicine, University of Sharjah, Sharjah, United Arab Emirates; 3 Institute for Nutritional Medicine, University of Lübeck, Lübeck, Germany; 4 Department of Biomedical Sciences, School of Veterinary Medicine, University of Pennsylvania, Philadelphia, PA USA; 5 Department of Pulmonary Medicine, Rashid Hospital, Dubai, United Arab Emirates; 6 Airway Research Center North, Member of the German Center for Lung Research (DZL), Lübeck, Germany; 7 Biomedically Informed Artificial Intelligence Laboratory (BIMAI-Lab), University of Sharjah, P.O. Box 27272, Sharjah, United Arab Emirates; 8 Division of Surgery and Interventional Science, University College London, London, WC1E 6BT UK; 9 Meakins-Christie Laboratories, McGill University, Montreal, QC Canada

**Keywords:** Asthma, Airway remodeling, Fibrosis, Interleukin-4 (IL-4), Interleukin-13 (IL-13), Dupilumab, Cell biology, Diseases, Immunology, Medical research, Pathogenesis

## Abstract

Airway fibrosis in severe asthma is a hallmark of disease pathology, yet the mechanisms driving fibroblast activation remain incompletely understood. While interleukin-4 (IL-4) and interleukin-13 (IL-13) are central to type 2 inflammation, their combined role in directly promoting human lung fibroblast-mediated fibrosis is not well defined. This study demonstrates that IL-4 and IL-13 act synergistically on asthmatic fibroblasts to potentiate a pro-fibrotic program via the shared interleukin-4 receptor alpha (IL-4Rα)/Signal Transducer and Activator of Transcription 6 (STAT6) axis. We found that diseased human lung fibroblasts (DHLFs) from asthmatic patients exhibited elevated expression of the type II receptor (IL-4Rα/IL-13Rα1) compared to normal human lung fibroblasts (NHLFs), priming them for activation. Consequently, combined IL-4/IL-13 stimulation synergistically enhanced STAT6 phosphorylation and robustly upregulated key extracellular matrix (ECM) components (collagen I/III, fibronectin) and regulators (matrix metalloproteinase-9 (MMP-9), tissue inhibitor of metalloproteinase-1/2 (TIMP-1/2). Furthermore, IL-4 specifically emerged as the dominant driver of fibroblast proliferation. Critically, the IL-4Rα-blocking antibody dupilumab effectively abrogated this pro-fibrotic response. These findings provide a direct mechanistic basis for targeting the IL-4Rα pathway to mitigate airway remodeling and potentially alter the progressive course of fibrosis in patients with severe, type 2-high asthma.

**Supplementary Information:**

The online version contains supplementary material available at 10.1038/s41598-026-54625-7.

##  Introduction

 Asthma is a major and growing global health burden, affecting over 300 million people and causing approximately 500,000 deaths in 2019^1^. It is defined as a chronic, heterogeneous inflammatory disorder characterized by persistent airway inflammation, bronchial hyperresponsiveness, and reversible airflow obstruction^2^. Despite significant advancements in therapies, delivery devices, and monitoring technologies, asthma prevalence and associated burdens continue to rise. A key challenge is the substantial heterogeneity in patient treatment responses^3^. Severe asthma which is characterized by its complex pathophysiology, diverse endotypes, inflammation, and resistance to conventional therapies, is a major contributor to these variations^4^. This severe phenotype is closely associated with dysregulated type 2 inflammation, orchestrated primarily by interleukins (IL)−4, IL-13, and IL-5^5^. These cytokines drive critical pathological features collectively perpetuating chronic inflammation and airway remodeling^6–8^.

Airway remodeling involves alterations in cellular and extracellular matrix (ECM) components in both large and small airways, epithelial cells detachment, proliferation of airway smooth muscle cells, and differentiation of fibroblasts into myofibroblasts^9^. In severe asthma airway remodeling is associated with high rate of goblet cell proliferation, sub-epithelial fibrosis, mucus secretion, and increased airway smooth muscle (ASM) mass^10–12^.

Among all these features sub-epithelial fibrosis is a well reported consequence of Th2 cytokine-dominated inflammatory response resulting in an increase in ECM protein deposition upon fibroblast activation^13^, leading to a stiffer and less elastic airway wall, making it more difficult for the airway to expand and contract^13,14^ as well as an overall narrowing of the airway lumen. ECM consist of fibrous proteins including collagen and elastin as well as structural or adhesive proteins like fibronectin and laminin which play an important role in supporting and maintaining tissue structure, in addition to other biological functions such as migration, and proliferation of fibroblast^15^. Therefore, equilibrium between synthesis and degradation of ECM components is required for the maintenance of its homeostasis.

Matrix metalloproteinases (MMPs) and their inhibitors tissue inhibitors of metalloproteinases (TIMPs) are likely to be the normal physiologically relevant mediators of ECM degradation^16,17^. Loss of co-ordination in the expression of proteinases and inhibitors is believed to generate tissue degradation in many diseases including asthma^18^. Inflammatory cytokines, including IL-4 and IL-13, have been reported to play a key role in this process^19,20^.

While both IL-4 and IL-13 are well-established mediators of inflammation in asthma, they also play significant roles in airway remodeling, despite sharing a common receptor subunit (IL-4Rα) and overlapping signaling pathways, IL-4 and IL-13 exhibit distinct biological functions in asthma pathogenesis^21^. IL-4 is more closely tied to the inflammatory cascades of type-2 asthma, driving Immunoglobulin E (IgE) switching and eosinophil recruitment^22^. Whereas, IL-13 is more strongly implicated in the remodeling processes, including mucus hypersecretion, fibrosis, and smooth muscle hypertrophy^22^. These unique and overlapping roles underscore the complex contributions of IL-4 and IL-13 in both the inflammatory and structural changes characteristic of asthma. Given their central involvement, IL-4 and IL-13 represent promising therapeutic targets for asthma management.

Dupilumab, a monoclonal antibody that simultaneously inhibits IL-4 and IL-13 activity by blocking the shared IL-4Rα receptor^23^, has been validated in both clinical trials and real-life settings as an effective agent for suppressing type 2 inflammation and improving asthma-related outcomes^23,24^. It contributes to asthma control through various mechanisms, including reducing the proliferation of type 2 immune cells such as Th2 and Innate lymphoid cells 2 (ILC2)^25,26^, decreasing the frequency of asthma exacerbations^27^, normalizing the expression of genes related to mucus production and tissue remodeling, and preventing eosinophil infiltration into the lungs^28^.

While IL-13 and IL-4 are established principal mediators of the structural alteration’s characteristic of airway remodeling in asthma, their direct contributions to remodeling and fibrosis require further investigation. Moreover, whether these cytokines exert synergistic or additive effects via their shared receptor (IL-4Rα) on airway fibroblasts in the asthmatic context remains unexplored.

Here, we investigate the dual effect of IL-4 and IL-13 on asthmatic fibroblast activation, including proliferation, ECM deposition, and MMP/TIMP expression. To our knowledge, this is the first study to demonstrate the synergistic pro-fibrotic role of IL-4 and IL-13 cytokines in asthma. It provides novel evidence that dual blockade of these cytokines using dupilumab is necessary to achieve broad inhibition of their direct fibrotic effects. These findings offer new mechanistic insights into fibrotic pathways and support the therapeutic potential of targeting both IL-4 and IL-13 simultaneously.

##  Materials and methods

### Cell culture and treatment

Human primary bronchial fibroblasts were obtained from Lonza, were isolated from non-smoking individuals with asthma and healthy patients. Table 1 defines the features of the subjects involved in this study, in which patients received one of the following treatments: Albuterol, Combivir, or Proventil. Cells were maintained in Lonza FGM Fibroblast Growth Medium (Lonza™ CC-3130) and/or Dulbecco’s Modified Eagle Medium F-12 (DMEM-F12) (Cat: 10565018, Gibco-Thermofisher) supplemented with 15% fetal bovine serum (FBS, Sigma Aldrich), 1% Penicillin/Streptomycin (Gibco) in a 37 °C humidified incubator (Thermo Scientific HERA cell 150i Carbon Dioxide Incubator) containing 5% CO2 and 21% oxygen. At ~ 80% confluency cells were seeded and starved overnight with FBS DMEM-F12 free medium then treated with 20 ng/ml of either rIL-4 (R&D, Cat: 204-IL) alone or 20ng/ml of rIL-13, (Prospec, Cat: cty-446-b) alone or 20 ng/ml of both rIL-4 and rIL-13, concentration was chosen based on previous work as in Saito, A. et al.^29^, for 24 and 48 h. Control wells were left untreated. To investigate the potential effect of dupilumab on fibroblast, cells were pre-treated with 68 µg/mL of dupilumab for 2 h, followed by stimulation with 20 ng/mL of rIL-4 and rIL-13 for 24 and 48 h. Dupilumab concentration was chosen based on dose optimization. Data were obtained from *n* = 3 independent donors for NHLFs and *n* = 3 independent donors for DHLFs. Experiments were repeated at least 3 times independently.

Primary human fibroblast cells from both healthy and asthmatic donors were obtained from Lonza. Since these cells were acquired from a commercial supplier and not collected directly from human participants, ethics committee approval was not required for this study. All donor-related information was provided by Lonza upon request.

### Immunofluorescence staining of human bronchial biopsy tissues

Formalin-fixed paraffin-embedded (FFPE) bronchial biopsy slides from non-asthmatic control subjects were obtained from the Biobank of the Quebec Respiratory Health Research Network, Canada (MUHC REB# BMB-02–039-t). Biopsies from asthmatic patients diagnosed according to American Thoracic Society (ATS) criteria and receiving treatment per Global Initiative for Asthma (GINA) guidelines were collected at the Severe Asthma Clinic, Pulmonary Medicine Department, Rashid Hospital, Dubai, UAE. Written informed consent was obtained from all participants. The study protocol was approved by the Dubai Scientific Research Ethics Committee (DSREC-11/2017_04). Biopsy collection and embedding were performed as previously described^30^. Sections from both healthy and asthmatic individuals were mounted on positively charged slides and used for hematoxylin and eosin (H&E) staining and immunofluorescence analysis. For immunostaining, tissue sections were deparaffinized, rehydrated, and subjected to heat-induced antigen retrieval using sodium citrate buffer (pH 6.0). Retrieval was performed in a microwave oven for 5 min at 750 W followed by 5 min at 450 W, after which slides were cooled at room temperature for 1 h. Tissue permeabilization was carried out using 0.05% Triton X-100 in PBS for 10 min at room temperature (RT). After rinsing with PBS, sections were blocked with 3% BSA in PBS-Tween for 1 h at RT. Primary antibodies anti-IL-4Rα (R&D Systems, MAB230), anti-IL-13Rα1 (Abcam, ab79277), and anti-vimentin (R&D Systems, MAB2105) were diluted 1:100 in blocking buffer and incubated overnight at 4 °C. Following primary antibody incubation, slides were washed with PBS and incubated with secondary antibodies Alexa Fluor^®^ 488-conjugated (Invitrogen) with an Excitation/Emission range of 499/520 nm and Alexa Fluor^®^ 594-conjugated (Thermo Fisher) with an Excitation/Emission range of 590/618 nm for 1 h at RT. Nuclei were counterstained with DAPI (Invitrogen, Cat. No. D1306) per manufacturer’s instruction. Fluorescent images were captured using a Nikon confocal microscope (Nikon, Tokyo, Japan), and mean fluorescence intensity was quantified using ImageJ (Fiji) software.

### Western blot

Post fibroblast stimulation with rIL-4, rIL-13, and both IL-4/IL-13 for 2- and 24-hours cells were collected and lysed using 30ul of 1x RIPA buffer (Abcam). Proteins were quantified using Pierce BCA protein assay kit (Cat# 23227, thermo scientific, USA). After that protein lysate was separated by 8–10% sodium dodecyl sulfate–polyacrylamide gel electrophoresis (SDS-PAGE) and transferred into polyvinylidene fluoride (PVDF) membrane using wet transfer method. Blots were blocked with 5% skimmed milk for 1 h and blotted with primary antibodies listed in Table 2, diluted at 1:1,000 with 5% BSA and kept overnight at 4 °C on the shaker. Blots were reacted with anti-mouse and anti-rabbit for 1 h at room temperature. Bands were detected using ECL kit (Bio-Rad, United States) and quantified by Bio-Rad Image Lab software (ChemiDoc™ Touch Gel and Western Blot Imaging System; Bio-Rad). The representative protein bands were either cropped sections from different blots or from different parts of the same blots. For blots that were re-probed, stripping was performed between each round of antibody probing. The uncropped blots are provided in the supplementary file with a clear delineation between bands and the molecular weights are indicated. GAPDH was used as the normalization control.

**Table 1:** Clinical characteristic of subject involved in this study.

Clinical characteristic	Healthy individuals (*n* = 3)	Asthmatic patients (*n* = 3)
Mean age (years)	58 (SD: 10.6, range: 25)	43 (SD: 1.6, range: 27)
Gender (M %: F %)	67: 33	33: 67
Ethnicity (Caucasian)	100%	100%
BMI (kg/m²)	39.9 (SD: 1.6, range: 4)	39.9 (SD: 4.7, range: 10)
Medications	None	Albuterol, Proventil, Combivir

**Table 2:** List of primary antibodies used in this study.

Protein	Manufacture, Cat#
IL-4Rα	R&D, MAB230
IL-13Rα1	Abcam, ab79277
MMP-2	CST, 87,809
MMP-9	CST, 13,667
TIMP-1	CST, 8946
TIMP-2	CST, 5738
Collagen-1A1	CST, 72026T
Collagen-3A1	CST, 98,908
Fibronectin	CST, 26,836
Lumican	Abcam, ab168348
Tenascin C	CST, 33,352
P-STAT6	CST, 9361
STAT6	CST, 5397
GAPDH	CST, 2118

### EdU proliferation assay

Upon rIL-4 and rIL-13 stimulation EdU proliferation assay that detects newly synthesized DNA by incorporating 5-ethynyl-2′-deoxyuridine (EdU), a thymidine analog, into replicating DNA during the S-phase of the cell cycle was performed and quantified by flow cytometry. Fibroblasts were seeded at 3 × 10^5^ cells/mL in 6 well plate till ~ 70% confluency. Cells were starved overnight using F12/Dulbecco’s Modified Eagle’s Medium (Gibco–Thermofisher) supplemented with 0.1% FBS and 1% Penicillin/Streptomycin (Sigma). Next, cells were stimulated with rIL-4 alone and rIL-13 alone and both together for 48 h, cells were harvested using 1x trypsin, washed and stained according to the manufacturer instructions of the used kit (Abcam, Cat: ab219801). In brief, cells were incubated with 10 µM EdU stain. Cells were then fixed and permeabilized before being further stained with an anti-EdU antibody conjugated with AlexaFluor (AF)−488. EdU positive cells were examined using flow cytometry (AccuriTM flow cytometers; BD). Three experimental runs were conducted to confirm representative data. Results were acquired using the BD FACS III Aria and analyzed using the FlowJo software.

### Statistical analysis

The results are expressed as mean ± SD from three independent experiments. Statistical comparisons between control and treated fibroblasts were conducted using one-way ANOVA test and for multiple comparison Tukey’s multiple comparison test was conducted using GraphPad Prism 8 (GraphPad, San Diego, CA, USA). p < = 0.05 was considered statistically significant. To evaluate whether the combined effects of IL-4 and IL-13 on fibroblast proliferation, ECM protein expression, and signaling responses were additive or synergistic, we compared the observed co-stimulatory response to the expected additive value derived from the individual cytokine effects. For each experimental outcome, the expected additive effect (E_add) was calculated as referred to Eq. (1):

1 $${\mathrm{E}}\_{\text{add }}={\text{ }}({\mathrm{rIL}} - {\mathrm{4}}\, - \,{\mathrm{Control}}){\text{ }}+{\text{ }}({\mathrm{rIL}} - {\mathrm{13}}\, - \,{\mathrm{Control}}){\text{ }}+{\text{ Control}}$$

where rIL-4 and rIL-13 represent the measured responses following stimulation with each cytokine alone, and Control refers to unstimulated baseline.

An effect was classified as additive when the combined treatment was greater than each single cytokine but less than the calculated E_add value. Synergistic interactions were defined as combined effects exceeding the E_add value. This approach follows established definitions of additive and non-additive interactions in drug/cytokine combination studies^31^.

## Results

### Type-2 receptor (IL-4Rα/IL-13Rα1) is positively expressed in healthy and asthmatic derived lung fibroblasts

**Fig. 1:**
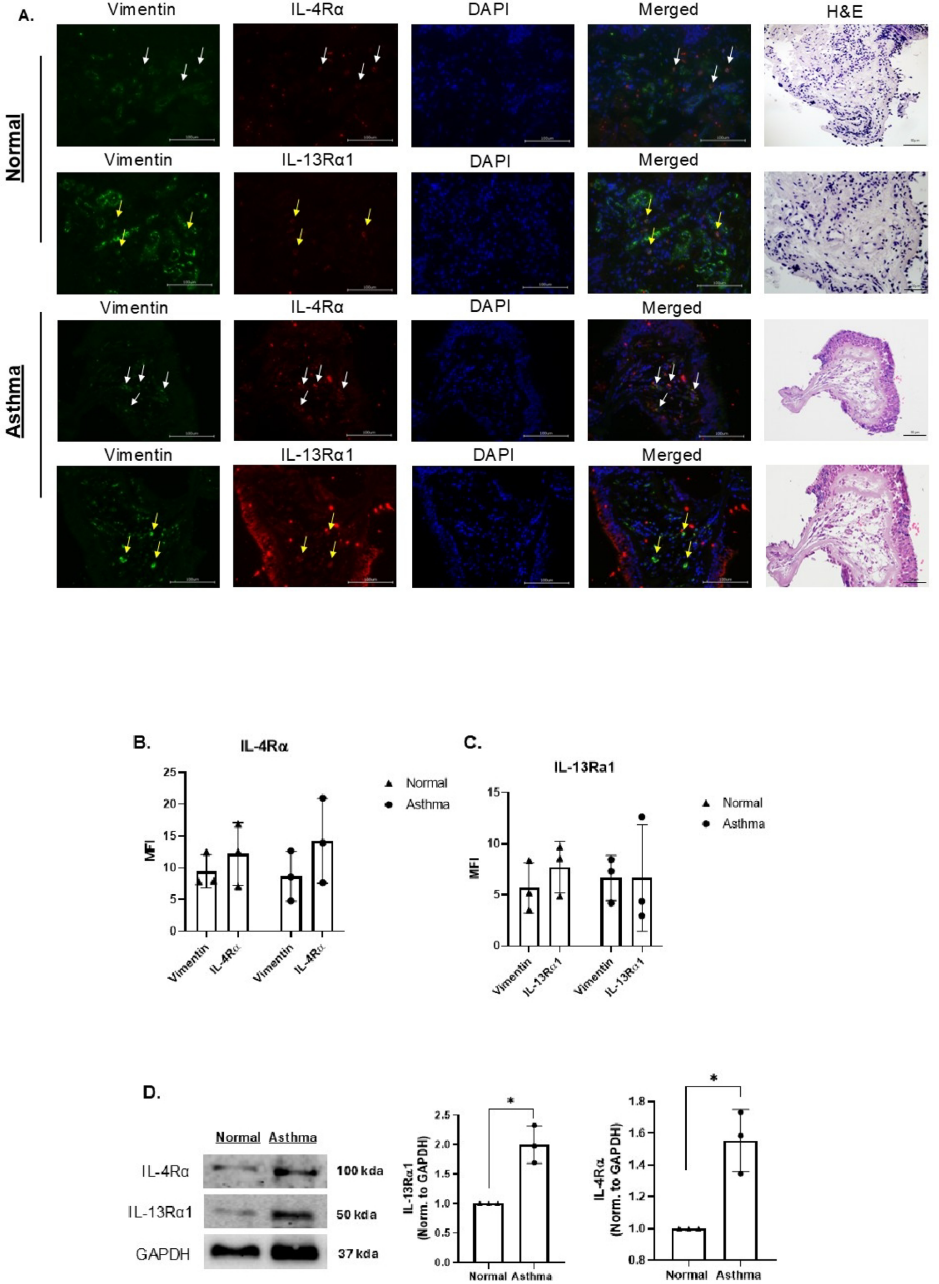
IL-4Rα and IL-13Rα1 expression in human lung fibroblasts. (**A**) H&E-stained sections of normal and asthmatic bronchial biopsies (right) (*n* = 3) taken at 20x and 40× magnification and immunofluorescence images showing Vimentin (green), IL-13Rα1 (red), IL-4Rα (red), and DAPI (blue) at 40× magnification in normal and asthmatic bronchial biopsy tissues. Yellow arrows indicate IL-13Rα1-positive fibroblasts and white arrows indicate IL-4Rα-positive fibroblasts. (**B**) Mean fluorescent intensity (MFI) of IL-4Rα and vimentin expression in bronchial biopsy tissues. (**C**) Mean fluorescent intensity (MFI) of IL-13Rα1 and vimentin expression in bronchial biopsy tissues. (**D**) Western blot analysis of IL-4Rα and IL-13Rα1 in fibroblasts from normal and asthmatic subjects; GAPDH was used as a loading control. The representative cropped blots/protein bands were obtained from different parts of the same gel. Uncropped western blot images are provided in supplementary file. Graphs show mean ± SD fold changes from at least three independent experiments. *p < 0.05 (unpaired two-tailed Student’s t-test).

Type-2 receptor is known to be expressed in both lymphocytes and non-hematopoietic cells. Therefore, the expression of Type-2 receptor was evaluated in human lung biopsies obtained from asthmatic patients compared to healthy control subjects. Immunofluorescence staining of IL-4Rα and IL-13Rα1 on fibroblast layers derived from healthy and asthmatic donors (Fig. 1A) showed no noticeable difference in expression levels or localization between the two groups. The mean fluorescence intensity (MFI) expression of IL-4Rα (Fig. 1B) and IL-13Rα1 (Fig. 1C) showed no significant diffrence in asthmatic fibroblasts compared to healthy cells. In contrast, Western blot analysis of protein lysates from lung derived fibroblast cells revealed a significant increased of the expression of both IL-4Rα (*P* = 0.0499) and IL-13Rα1 (*P* = 0.0240) in the asthmatic subjects compared to the healthy controls (Fig. 1D). These findings suggest that although immunofluorescence did not detect differential expression at the cellular layer level, Western blotting revealed an upregulation of total receptor protein in fibroblasts from asthmatic patients.

### rIL-4 and rIL-13 induce Type-2 receptor expression, activation and lung fibroblast proliferation

Given that asthma patients exhibit elevated protein expression of the IL-4/IL-13 receptor complex, we explored the possible positive feedback loop of rIL-4 and/or rIL-13 on the expression levels of IL-4Rα and IL-13Rα1. Asthmatic fibroblast cells were stimulated under three different conditions: rIL-4, rIL-13, and a combination of both for 24 h.

**Fig. 2:**
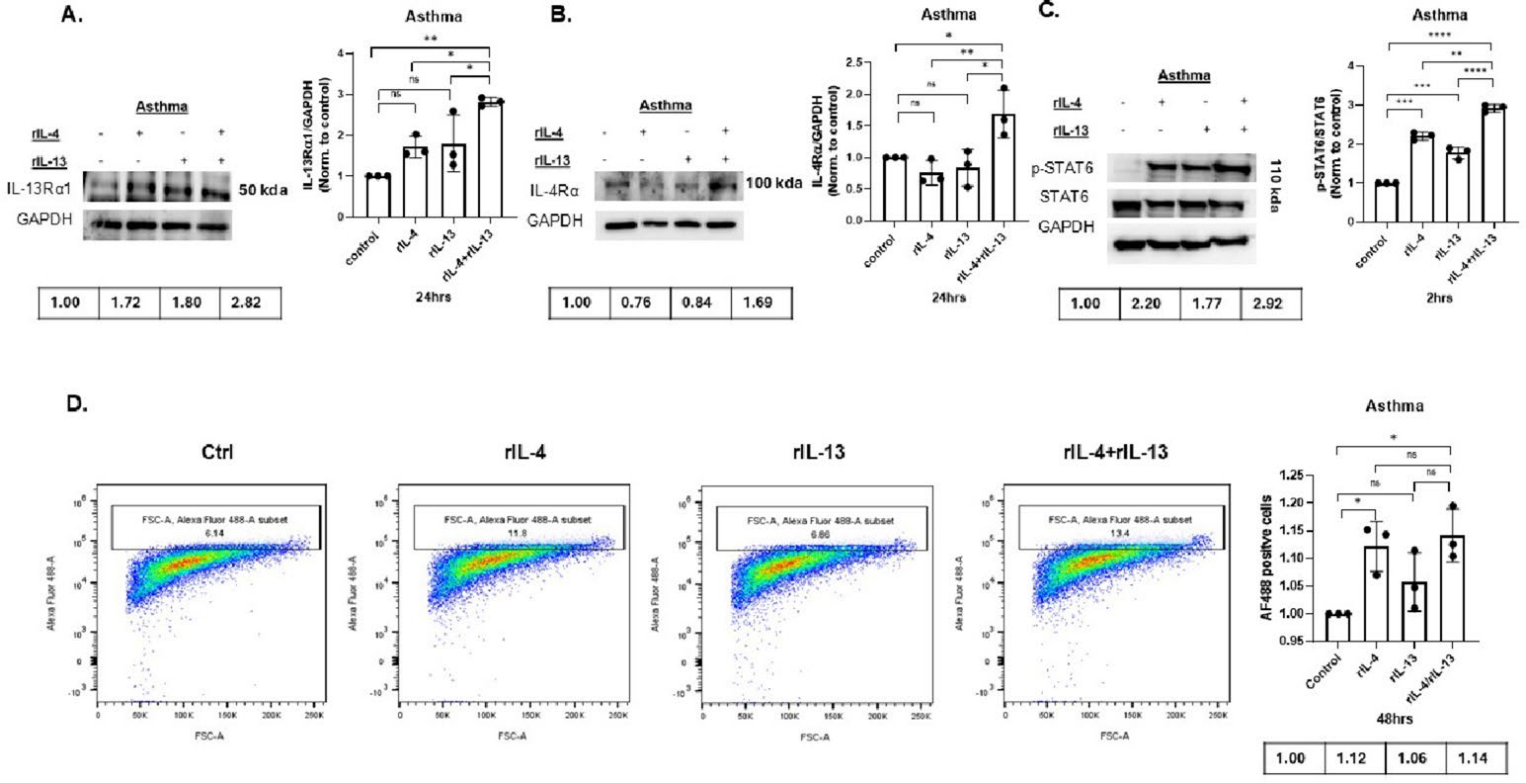
Effects of rIL-4 and rIL-13 stimulation on type 2 receptor expression and functional activity in asthmatic airway fibroblasts. Expression of (**A**) IL-13Rα1, (**B**) IL-4Rα, at 24 h and (**C**) phosphorylated STAT6 at 2 h in asthmatic lung fibroblasts treated with rIL-4, rIL-13, or both. Immunoblot analysis was done, and data represents SD from at least three independent experiments, normalized to untreated controls. GAPDH was used as a loading control. The representative cropped blots/protein bands of IL-4Rα and IL-13Rα1 were obtained from different gels. Uncropped western blot images are provided in supplementary file. Statistical analysis was conducted using one-way ANOVA. *p < 0.05, **p < 0.01, ***P > 0.001, ****P > 0.0001. (**D**) Proliferation assay of asthmatic lung fibroblasts was performed after 48-hour stimulation under the same conditions. EdU stained cells were identified as AF488 + cells as they label the proliferating cells with newly synthesized DNA. Statistical analysis was conducted using one-way ANOVA. *p < 0.05.

For IL-13Rα1 (Fig. 2A) no significant increase was observed for either rIL-4 and rIL-13 alone relative to control, with mean values of 1.72 ± 0.25 and 1.80 ± 0.67, respectively. In contrast, the combined treatment markedly enhanced expression (2.82 ± 0.10), exceeding the effect of either cytokine alone and surpassing the expected additive value (E_add = 2.52). Similarly, rIL-4 (0.76 ± 0.20) or rIL-13 (0.84 ± 0.29) showed no significant effect on the expression of IL-4Rα (Fig. 2B) relative to control (1.00 ± 0.00). However, co-treatment with both cytokines significantly increased IL-4Rα expression (1.69 ± 0.38), with an effect greater than that predicted by additivity (E_add = 0.60).

To further evaluate downstream signaling, we assessed STAT6 phosphorylation (Fig. 2C) post 2 h stimulation as a marker of cellular activation. rIL-4 (2.20 ± 0.10) and rIL-13 (1.77 ± 0.15) significantly increased phosphorylation compared to control (1.00 ± 0.00). The combination (2.92 ± 0.09) further elevated p-STAT6 relative to either cytokine alone reaching closely to the expected additive level (E_add = 2.97).

Given that both cytokines enhanced receptor expression and STAT6 downstream signaling activation, and consistent with previous studies showing that stimulation with IL-4 or IL-13 alone promotes fibroblast activation and proliferation via a STAT6-dependent pathway, we next assessed the effect of rIL-4 and rIL-13 in combination on fibroblast proliferation post 48 h (Fig. 2D), measured as the proportion of AF488⁺ cells. Specifically, Kriebel et al.^32^ tested IL-4 alone and Ingram et al.^33^ tested IL-13 alone. rIL-4 (1.12 ± 0.05) increased proliferation relative to control (1.00 ± 0.00), while rIL-13 (1.06 ± 0.05) had no detectable effect. The combined treatment (1.14 ± 0.05) produced a proliferation level similar to rIL-4 alone and did not approach the expected additive value (2.18), indicating that combination of rIL-4 and rIL-13 yields only a small, non-additive effect on fibroblast proliferation. Additionally, rIL-4 and rIL-13 showed no apoptotic effect in those patients (see Supplementary Fig. S1 online) Overall, these findings indicate that rIL-4 and rIL-13 synergistically upregulated both receptors, enhancing cellular responsiveness. This was reflected in a stronger activation of downstream STAT6 signaling, which closely aligned with additive expectations, but this did not translate into an effect on fibroblast proliferation.

###  rIL-4 and rIL-13 induce ECM protein expression in asthmatic derived lung fibroblast

Since both cytokines demonstrated an effect on fibroblast activation and given that activated fibroblasts are the predominant cell type in the fibrotic layer and play a central role in ECM production^34^, we evaluated a panel of ECM proteins and matrix regulatory enzymes by western blot assay following 24 h of rIL-4 and rIL-13 stimulation.

**Fig. 3:**
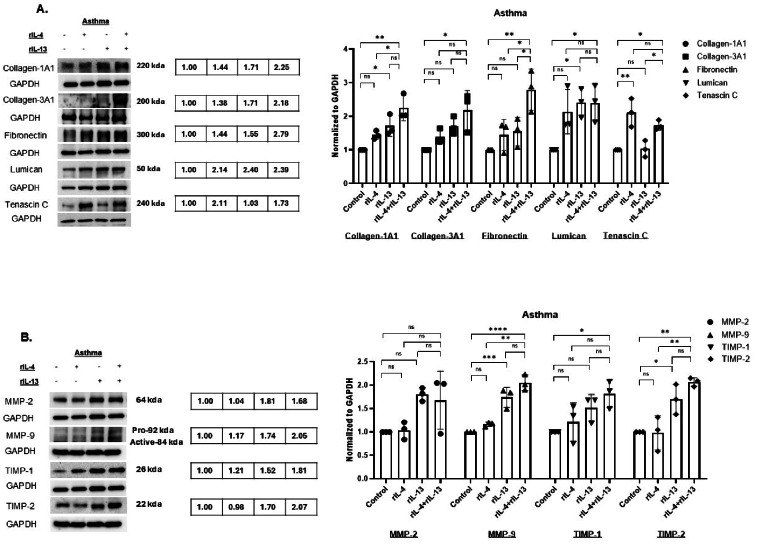
Effects of rIL-4 and rIL-13 Stimulation on ECM and MMP/TIMP Expression in Asthmatic Lung Fibroblasts. Expression of (**A**) ECM proteins: Collagen I, Collagen III, fibronectin, lumican, and tenascin C. (**B**) matrix remodeling proteins: MMP-2, MMP-9, TIMP-1, and TIMP-2 at 24 h in asthmatic lung fibroblasts treated with rIL-4, rIL-13, or both. Immunoblot analysis was done, and data represents SD from at least three independent experiments, normalized to untreated controls. The representative cropped blots/proteins bands of MMP-2 and TIMP-1 were obtained from different parts of the same gel as well as MMP-9 and TIMP-2, other protein bands were obtained from different gels. GAPDH was used as a loading control. Uncropped western blot images are provided in supplementary file. Statistical analysis was performed using one-way ANOVA; *p < 0.05, **p < 0.01, ***P > 0.001, ****P > 0.0001.

First, we examined collagens, which are strongly associated with asthma severity^35^. As shown in Fig. 3A for collagen-1A1, rIL-13 (1.71 ± 0.31) showed a significant increase in protein expression compared to control (1.00 ± 0.00) whereas no effect was observed in response to rIL-4. Combination of both (2.25 ± 0.38) further enhanced the expression above than the expected additive value (E_obb = 2.15). However, collagen-3A1 expression was not affected by rIL-4 (1.38 ± 0.21) or rIL-13 (1.71 ± 0.26) while the combination of both (2.18 ± 0.56) did induce a significant effect than single cytokines compared to the control (E_add = 2.09). Similarly, fibronectin expression was not affected by rIL-4 (1.44 ± 0.45) or rIL-13 (1.55 ± 0.42) in comparison to the control, yet the expression induced by the combination of both cytokines (2.79 ± 0.61) was significantly increased and exceeded the expected additive value (E_obb = 1.99). In contrast, lumican was markedly upregulated by rIL-13 only (2.40 ± 0.43), while rIL-4 and rIL-13 combination (2.39 ± 0.55) did not exceed rIL-13 alone compared to the control, falling short of additive expectation (E_obb = 3.54). For Tenascin-C, rIL-4 strongly increased expression (2.11 ± 0.42), rIL-13 had no effect (1.03 ± 0.25), and the combination (1.73 ± 0.14) resulted in an intermediate increase that was below rIL-4 alone, suggesting a lack of additive or synergistic regulation.

MMPs, along with their endogenous inhibitors, TIMPs are key regulators of ECM proteins. The delicate balance between MMPs activities and TIMPs expression governs ECM turnover and structural integrity, and its disruption contributes to pathological tissue remodeling observed in chronic inflammatory diseases such as asthma^36^. To further explore how type 2 cytokines influence this regulatory axis in fibroblasts, we assessed the expression of MMPs and TIMPs (Fig. 3B) following stimulation with rIL-4, rIL-13, and their combination.

For MMP-2, no significant changes were observed by any of these conditions. MMP-9 was not affected by rIL-4 while strongly induced by rIL-13 (1.74 ± 0.22); the combination (2.05 ± 0.17) further elevated expression above the predicted additive level (E_obb = 1.91). TIMP-1 was only induced upon the combination of both cytokines (1.81 ± 0.29) exceeding the additive expectation (E_obb = 1.73). Similarly, TIMP-2 was predominantly induced by rIL-13 (1.70 ± 0.32), with no effect of rIL-4 alone (0.98 ± 0.37); the combination (2.07 ± 0.09) further increased the expression again exceed the predicted additive value (E_add = 1.72).

Overall, the data indicates that IL-4 and IL-13 together exert a stronger effect on fibroblast ECM regulation than either cytokine alone. Notably, the combination produced synergistic increases in collagen-1A1, collagen-3A1, and fibronectin expression, as well as in the regulatory proteins MMP-9, TIMP-1, and TIMP-2. These findings underscore the potential value of therapeutic approaches targeting both cytokine pathways to more effectively mitigate fibrotic progression in asthma.

### Dupilumab inhibits IL-4 and IL-13 signaling via IL-4Rα binding and reduces fibroblast proliferation

To investigate whether interfering with the IL-4Rα and IL-13Rα1 signaling mitigates the potential fibrotic effects of IL-4 and IL-13, cells were first treated with dupilumab, a specific anti-IL-4Rα antibody and subsequently stimulated with both cytokines. Receptor expression, signaling pathway activation, and cellular proliferation were then evaluated.

**Fig. 4:**
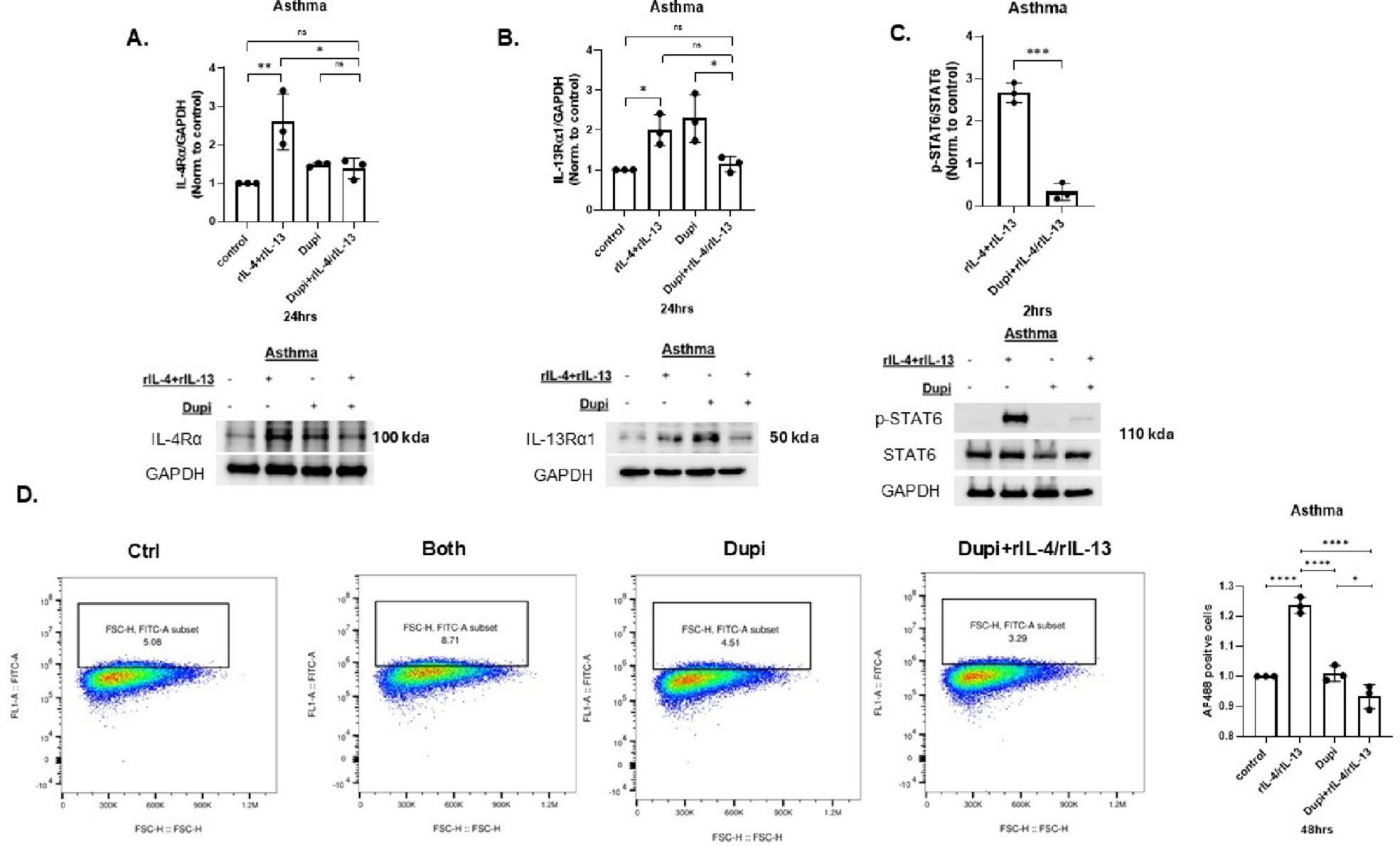
Dupilumab blocks IL-4/IL-13-induced receptor expression, STAT6 activation, and fibroblast proliferation. Expression of (**A**) IL-4Rα, (**B**) IL-13Rα1 (**C**) STAT6 phosphorylation in asthmatic derived lung fibroblasts treated with dupilumab 2 h prior 24 h and 2 h stimulation with IL-4 and IL-13. Western immunoblot analysis was done, and data represent mean ± SD from at least three independent experiments, normalized to untreated controls. The representative cropped blots/protein bands were obtained from different gels. GAPDH was used as a loading control. Uncropped western blot images are provided in supplementary file. Statistical analysis was performed using one-way ANOVA; *P > 0.05, **P > 0.01, ***P > 0.001. (**D**) Proliferation assays of asthmatic lung fibroblasts were performed after 48-hour stimulation under the same conditions of dupilumab treatment. EdU stained cells were identified as AF488 + cells as they label the proliferating cells with newly synthesized DNA. Statistical analysis was performed using one-way ANOVA; *P > 0.05, ****p > 0.0001.

Our western blot data showed that pre-treatment with dupilumab significantly dampens the enhanced response of IL-4Rα1 protein expression (*P* = 0.0212) observed with rIL-4 and rIL-13 co-stimulation (Fig. 4A). In contrast, IL-13Rα1 expression showed no significant changes following dupilumab treatment (Fig. 4B). Notably, dupilumab completely inhibited STAT6 phosphorylation (*P* = 0.0002), which was otherwise strongly induced by rIL-4/rIL-13 co-stimulation, indicating an effective blockade of IL-4Rα downstream signaling (Fig. 4C). Although rIL-13 alone or in combination with rIL-4 showed non-additive proliferative effect, blocking the shared IL-4Rα receptor with dupilumab significantly reduced IL-4 induced fibroblast proliferation (*P* < 0.0001), as evidenced by a decrease in AF488⁺ cells (Fig. 4D). These findings demonstrate that dupilumab effectively inhibits IL-4Rα-mediated signaling and IL-4 driven fibroblast proliferation, highlighting its potential therapeutic role in limiting fibroblast-driven airway remodeling in asthma.

###  Dual IL-4/IL-13 blockade attenuates ECM protein expression in asthmatic lung fibroblasts

To further evaluate the impact of the blockade of IL-4Rα/IL-13Rα1 signaling with dupilumab on fibrotic capabilities of fibroblasts, we assessed further the expression of ECM molecules.

**Fig. 5:**
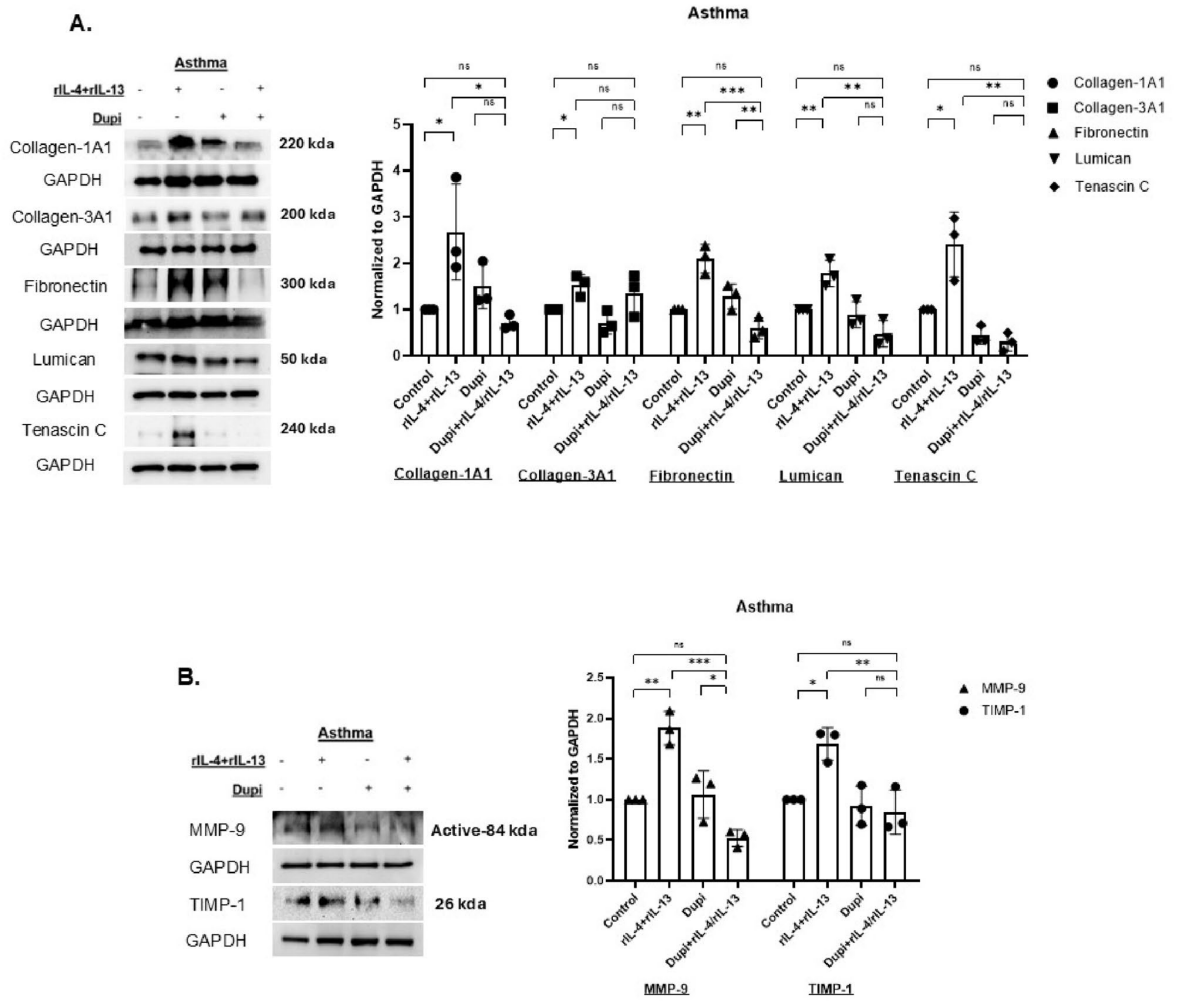
IL-4/IL-13 blockade impact on matrix protein expression and MMP/TIMP homeostasis in asthmatic derived fibroblasts. Expression of (**A**) ECM proteins: Collagen 1A1, Collagen 3A1, fibronectin, lumican, and tenascin C. (**B**) Matrix remodeling proteins MMP-9, and TIMP-1 in asthmatic derived lung fibroblasts treated with dupilumab 2 h prior 24 h stimulation with IL-4 and IL-13. Data represent mean ± SD from at least three independent experiments, normalized to untreated controls. The representative cropped blots/protein bands were obtained or from different gels except lumican and tenascin C from different parts of the same gel. GAPDH was used as a loading control. Uncropped western blot images are provided in supplementary file. Statistical analysis was performed using one-way ANOVA; *p < 0.05, **p < 0.01, ***p < 0.001.

Dupilumab dampened the combined effect of IL-4 and IL-13 by significantly reducing collagen I (*P* = 0.0132) and fibronectin (P = > 0.0001) expression compared to co-stimulated group in the absence of dupilumab (Fig. 5A) whereas no reduction in Collagen III post dupilumab treatment could be observed, suggesting that Collagen III expression may be partially regulated through IL-4Rα-independent mechanisms or alternative signaling pathways. Additionally, although IL-4 appeared to be the dominant regulator of tenascin C and IL-13 for lumican, dupilumab significantly reduced the expression of both ECM proteins (lumican, *P* = 0.0009 and tenascin C, *P* = 0.0006) upon IL-4Rα blockade. This suggests that both cytokines may converge on IL-4Rα-dependent signaling pathways, and that IL-4Rα is essential for the downstream regulation of tenascin C and lumican, even when one cytokine is the dominant inducer.

To further determine the contribution of IL-4Rα signaling, we assessed MMP-9 and TIMP-1 following dupilumab treatment (Fig. 5B), MMP-9 expression was significantly reduced compared with IL-4 + IL-13 stimulation, indicating that IL-4Rα contributes to MMP-9 induction. In parallel, TIMP-1 expression was decreased, demonstrating that the IL-4Rα pathway is also required for maximal TIMP-1 upregulation.

These findings demonstrate that IL-4Rα blockade with dupilumab broadly limits fibroblast-driven ECM remodeling. This mechanism provides a potential explanation for the clinical benefits observed in patients with asthma, where dupilumab not only reduces inflammation but may also help prevent long-term fibrotic changes contributing to disease progression.

## Discussion

Asthma is a chronic inflammatory airway disease characterized by variable airflow obstruction, bronchial hyperresponsiveness, and structural airway remodeling. Airway fibrosis is a key component of this remodeling and contributes to progressive loss of lung function through disrupted ECM turnover and excessive ECM deposition, leading to airway wall thickening and airflow limitation^9,37^.

Among the type-2 cytokines involved, IL-13 is widely recognized as a major pro-fibrotic mediator, whereas IL-4 exerts context-dependent effects on fibrosis^13,29,38^. Together, these cytokines regulate fibroblast activation and airway remodeling, which may explain why simultaneous blockade of IL-4 and IL-13 via IL-4Rα inhibition provides broader suppression of type-2 inflammation and structural remodeling than targeting IL-13 alone^28,39–41^.

This study aimed to investigate the potential fibrotic effect of IL-4 and IL-13 individually and in combination on asthmatic fibroblasts and to evaluate whether IL-4Rα blockade with dupilumab could attenuate these profibrotic responses. To our knowledge, this is among the first studies to assess dual pro-fibrotic effects of IL-4 and IL-13 as well as dupilumab direct effects on primary fibroblasts derived from asthmatic patients, offering novel insights into structural remodeling beyond immune modulation.

IL-4Rα is the shared subunit of type II receptor for IL-4 and IL-13, and is expressed on immune cells^42–44^ as well as on structural cells, including fibroblasts^45,46^. Previous studies have demonstrated that IL-4 exert a higher binding affinity with IL-4Rα, while IL-13 exhibits lower to moderate affinity for both IL-4Rα and IL-13Rα1 suggesting that IL-4 and IL-13 may compete for the availability of ligand-binding subunits in cells expressing the type II receptor^47^.

In our study, fibroblasts derived from asthmatic lung tissue exhibited higher expression of IL-4Rα and IL-13Rα1 compared with healthy controls, indicating increased responsiveness to IL-4 and IL-13. Although no significant difference in surface receptor expression was detected by immunofluorescence, Western blot analysis revealed increased total protein levels in asthmatic fibroblasts. This discrepancy may reflect methodological differences between the two techniques, as Western blotting detects denatured proteins and linear epitopes, whereas immunofluorescence identifies proteins in their native conformation^48^. Co-stimulation with IL-4 and IL-13 further amplified receptor expression and enhanced downstream signaling, evidenced by markedly increased STAT6 phosphorylation, a key mediator of IL-4Rα activation in fibroblasts^47^. Notably, this response exceeded that induced by either cytokine alone, supporting a synergistic effect of combined IL-4/IL-13 signaling on fibroblast activation. In contrast, IL-4Rα blockade reduced receptor expression and abolished STAT6 phosphorylation, highlighting the central role of IL-4Rα in mediating these responses. These findings align with the established paradigm that IL-4 and IL-13 converge on a common signaling cascade culminating in STAT6 activation^49,50^, which remains the predominant mediator of IL-4/IL-13 signaling in fibroblasts despite reports of alternative pathways such as STAT3 in other cell types^47^.

Our findings further clarify the role of STAT6 in IL-4/IL-13 mediated fibroblast proliferation^51,52^. While previous studies suggested that these cytokines promote proliferation mainly in mild asthma but not in severe disease^51^, we observed a consistent proliferative effect of IL-4 in fibroblasts derived from asthma patients. In contrast, IL-13 alone did not significantly induce proliferation, consistent with reported variability in IL-13 responsiveness^51^. Whereas combination of both cytokines exerted a significant effect, but it did not exceed the response elicited by rIL-4 alone. Notably, blockade of IL-4Rα reduced fibroblast proliferation back to baseline levels, indicating that IL-4Rα/STAT6 signaling is a key driver of fibroblast proliferation and airway remodeling in asthma, providing mechanistic support for the clinical efficacy of IL-4Rα targeting therapies.

Previous studies have shown that increased fibroblast numbers and activation contribute to excessive ECM synthesis and deposition, leading to airway wall thickening and progressive fibrotic remodeling^53^. To better understand the contribution of IL-4 and IL-13 to this process, we evaluated their effects on ECM protein expression. Although limited studies have examined their role in ECM regulation in asthma, both cytokines have been implicated in fibrotic processes. IL-13 has been reported to induce collagen production in fibrotic diseases and to regulate collagen expression in airway fibroblasts through an MMP-2 dependent mechanism^54–56^. IL-4 has also been shown to promote ECM-related gene expression and collagen synthesis in fibroblasts^57,58^. Our findings demonstrate that IL-4 and IL-13 synergistically promote ECM protein expression in asthmatic fibroblasts. IL-13 alone significantly upregulated collagen I, while co-stimulation with IL-4 and IL-13 further enhanced collagen 1, collagen III, and fibronectin expression surpassing the additive expectation, indicating a synergistic interaction. In addition to collagens and fibronectin, we examined the regulation of non-collagenous ECM proteins involved in airway remodeling. Tenascin-C, a remodeling-associated damage-associated molecular pattern elevated in asthmatic airways and responsive to anti-inflammatory therapy^59,60^, and lumican, which contributes to collagen organization and airway mechanics and shows increased subepithelial deposition in asthma^61^. Previous studies have reported that IL-4 and IL-13 induce tenascin-C expression in lung fibroblasts and bronchial epithelial cells^62,63^. However, there is no current evidence indicating that these cytokines regulate lumican in asthma. In our study, co-stimulation increased both tenascin-C and lumican expression without evidence of synergistic induction. Interestingly, lumican expression was predominantly driven by IL-13, whereas tenascin-C responded more strongly to IL-4, suggesting cytokine-specific regulation of distinct ECM components. Together, these findings indicate that IL-4 and IL-13 promote a broad profibrotic ECM program in asthmatic fibroblasts. This underscores the complexity of IL-4/IL-13 mediated fibroblast activation and identifies combined blockade of these cytokines as a potentially more effective strategy to mitigate airway remodeling than inhibition of either alone.

Consistent with this, dupilumab treatment reversed the induction of collagen I and fibronectin and significantly reduced tenascin-C and lumican expression, supporting the central role of IL-4/IL-13 signaling in ECM production and remodeling. These findings align with previous studies showing that dupilumab attenuates fibrotic responses in steroid-hyporesponsive murine models of asthma^64^. In contrast, collagen III expression was not affected by dupilumab, suggesting regulation through alternative pathways. Indeed, collagen III expression in asthma has been linked primarily to TGF-β/Smad signaling, although other mediators such as nerve growth factor (NGF) may also contribute independently of TGF-β1^65,66^.

In asthma, IL-13 and IL-4 shift the ECM balance toward structural remodeling by upregulating both MMPs and their inhibitor TIMPs. Previously studies demonstrated that IL-13 induces MMP-9 and MMP-2 expression in airway fibroblasts, while MMP-9 facilitates the activation of latent TGF-β1, amplifying fibrotic remodeling^56,67^. Genetic deletion studies in MMP-9 and MMP-12 deficient mice further highlight the role of these proteases in IL-13 driven airway remodeling and fibrosis^67^. Concurrently, IL-13 augments TIMP-1 production both directly and in cooperation with TGF-β1 in fibroblasts and airway epithelial cells^68,69^. Moreover, IL-4 and IL-13 have been shown to induce ASM contractility through MMP-1 production^70^. Less evidence observed on IL-4 participating in MMPs and TIMPs activity in asthma.

Our results highlight distinct and cooperative roles of IL-4 and IL-13 in regulating the MMPs and TIMPs balance in asthma. IL-13 alone significantly increased MMP-9 and TIMP-2 expression, supporting its dominant role in matrix remodeling. In contrast, IL-4 alone did not significantly alter the expression of the MMPs or TIMPs examined, suggesting a more limited or context-dependent effect. Interestingly, co-stimulation induced broader changes, with significant upregulation of MMP-9, TIMP-1, and TIMP-2, but not MMP-2, indicating a synergistic interaction that favors ECM accumulation. Importantly, dupilumab treatment reduced MMP-9 expression and restored TIMP-1 levels to baseline. Together, these findings suggest that IL-13 is the primary driver of MMP/TIMP dysregulation, while IL-4 amplifies these effects under co-stimulatory conditions, and that dual inhibition by dupilumab may help restore matrix balance and limit airway remodeling.

The pro-fibrotic role of IL-4 and IL-13 in asthma and the therapeutic potential of dupilumab aligns with recent in vivo evidence demonstrating the therapeutic benefits of IL-4Rα blockade. A recent study using asthma mouse model showed that intranasal dupilumab significantly improved steroid responsiveness, reduced airway inflammation and tissue remodeling^64^. While the clinical efficacy of dupilumab has largely been attributed to suppression of type 2 inflammation, our findings extend this understanding by demonstrating that IL-4Rα/STAT6 signaling in fibroblasts directly contributes to airway remodeling through enhanced proliferation and ECM deposition. The ability of IL-4Rα blockade to normalize fibroblast proliferation, reduce profibrotic ECM expression, and restore MMP/TIMP balance suggests that dupilumab may exert both anti-inflammatory and anti-remodeling effects.

These mechanistic insights also align with the increasing recognition that clinical remission is an achievable treatment goal in asthma with biologic therapies. Remission is characterized not only by symptom control and reduced exacerbations, but also by decreased corticosteroid dependence and preservation of lung function. This is particularly relevant because airway remodeling contributes to steroid resistance and persistent airflow limitation, which are often only partially reversible with conventional therapies. By targeting structural remodeling pathways in fibroblasts, dupilumab may therefore help explain the sustained clinical improvements observed in some patients.

Consistent with this, recent clinical studies report that approximately 19% of asthma patients achieve clinical remission after 12 months of dupilumab therapy^71^. These findings suggest that long-term IL-4Rα inhibition may interrupt the inflammatory-remodeling cycle and potentially exert disease-modifying effects. Integrating IL-4Rα targeted therapy with conventional treatments may therefore improve clinical outcomes by addressing both inflammatory and fibrotic mechanisms underlying asthma progression.

Overall, this study provides mechanistic evidence that IL-4 and IL-13 act independently and synergistically to promote fibroblast proliferation, ECM deposition, and MMP/TIMP imbalance, thereby contributing to airway remodeling in asthmatic patients. By demonstrating that IL-4Rα/STAT6 signaling is central to these processes, and that dupilumab attenuates fibroblast activation and profibrotic responses, our findings extend the therapeutic relevance of IL-4Rα blockade beyond inflammation to include direct modulation of airway remodeling. However, further studies are needed to elucidate the downstream molecular mechanisms and signaling networks involved in IL-4Rα/STAT6-mediated extracellular matrix remodeling.

A limitation of this study is the relatively small sample size, which may limit the generalizability of the results. Nonetheless, the use of well-characterized asthmatic fibroblasts enhances the internal validity of our findings, and larger studies are warranted to confirm the dual role of IL-4 and IL-13 in airway remodeling.

## Supplementary Information

Below is the link to the electronic supplementary material.

Supplementary Material 1

## Data Availability

Not applicable.
