# Supplementary material for: Dupilumab attenuate IL-4 and IL-13 synergistic effect on airway remodeling in asthma via blocking IL-4Rα/STAT6 axis
Source: Sci Rep. 2026 May 30;16:24753. doi: 10.1038/s41598-026-54625-7 (PMC13458785; doi:10.1038/s41598-026-54625-7)
Supplement: Supplementary file 1 [file 41598_2026_54625_MOESM1_ESM.pdf]

Dupilumab attenuate IL-4 and IL-13 synergistic effect on airway remodeling in asthma via blocking IL-4Rα/STAT6 axis

Lina Sahnoon[1,2,3], Rola Abujabal[1,2], Tasneem M. Alanta[1], Bushra Mdkhana[1], Ronald Olivenstein[9], Ellen Puré[4], Bassam Mahboub[5*], Yves Laumonnier[3,6], Rifat Hamoudi[1,2,7,8], Khuloud Bajbouj[4*], Qutayba Hamid[1,2,9*†]

1. Research Institute of Medical and Health Sciences, University of Sharjah, Sharjah P.O. Box 27272, United Arab Emirates.
2. College of Medicine, University of Sharjah, Sharjah, United Arab Emirates.
3. Institute for Nutritional Medicine, University of Lübeck, Lübeck, Germany.
4. Department of Biomedical Sciences, School of Veterinary Medicine, University of Pennsylvania, Philadelphia, Pennsylvania, USA.
5. Department of Pulmonary Medicine, Rashid Hospital, Dubai, United Arab Emirates.
6. Airway Research Center North, Member of the German Center for Lung Research (DZL), Lübeck, Germany.
7. Biomedically Informed Artificial Intelligence Laboratory (BIMAI-Lab), University of Sharjah, Sharjah P.O. Box 27272, United Arab Emirates.
8. Division of Surgery and Interventional Science, University College London, London WC1E 6BT, UK.
9. Meakins-Christie Laboratories, McGill University, Montreal, Québec, Canada.

Email addresses:

Lina Sahnoon (L.S): U22105744@sharjah.ac.ae
Rola Abujabal (R.A): U21105877@sharjah.ac.ae
Tasneem M. Alanta (T.M.A): Tasneemalanta@outlook.com
Bushra Mdkhana (B.Md): bmdkhana@sharjah.ac.ae
Ronald Olivenstein (R.O): ronald.olivenstein@mcgill.ca
Ellen Puré (E.P): Epure@upenn.edu
Bassam Mahboub (B.M): drbassam_mahboub@yahoo.com
Yves Laumonnier (Y.L): Yves.Laumonnier@uksh.de
Rifat Hamoudi (R.H): rhamoudi@sharjah.ac.ae
Khuloud Bajbouj (K.B): kbajbouj@upenn.edu
Qutayba Hamid (Q.H): qalheialy@sharjah.ac.ae

Footnote:
† Qutayba Hamid passed away on January 11, 2026.
Corresponding author: Bassam Mahboub, Bassam Mahboub, drbassam_mahboub@yahoo.com.

* Equal senior corresponding authors.
Correspondence: Qutayba Hamid, qalheialy@sharjah.ac.ae
Bassam Mahboub, drbassam_mahboub@yahoo.com
Khuloud Bajbouj, kbajbouj@upenn.edu

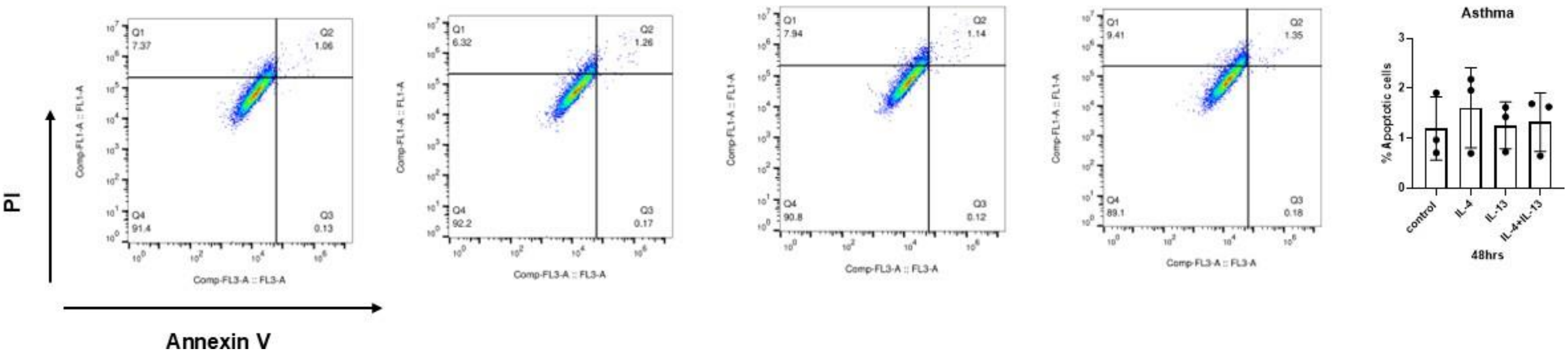

Supplementary Figure.S1 IL-4 and IL-13 exert no effect on apoptosis in asthmatic lung fibroblast. The Annexin V/PI flow cytometry-based method used to assess the percentage of apoptotic and pro-apoptotic post 48hrs treatment with rIL-4, rIL-13, or both in asthma patients. Statistical analysis was conducted using one-way ANOVA.

Figure 1D

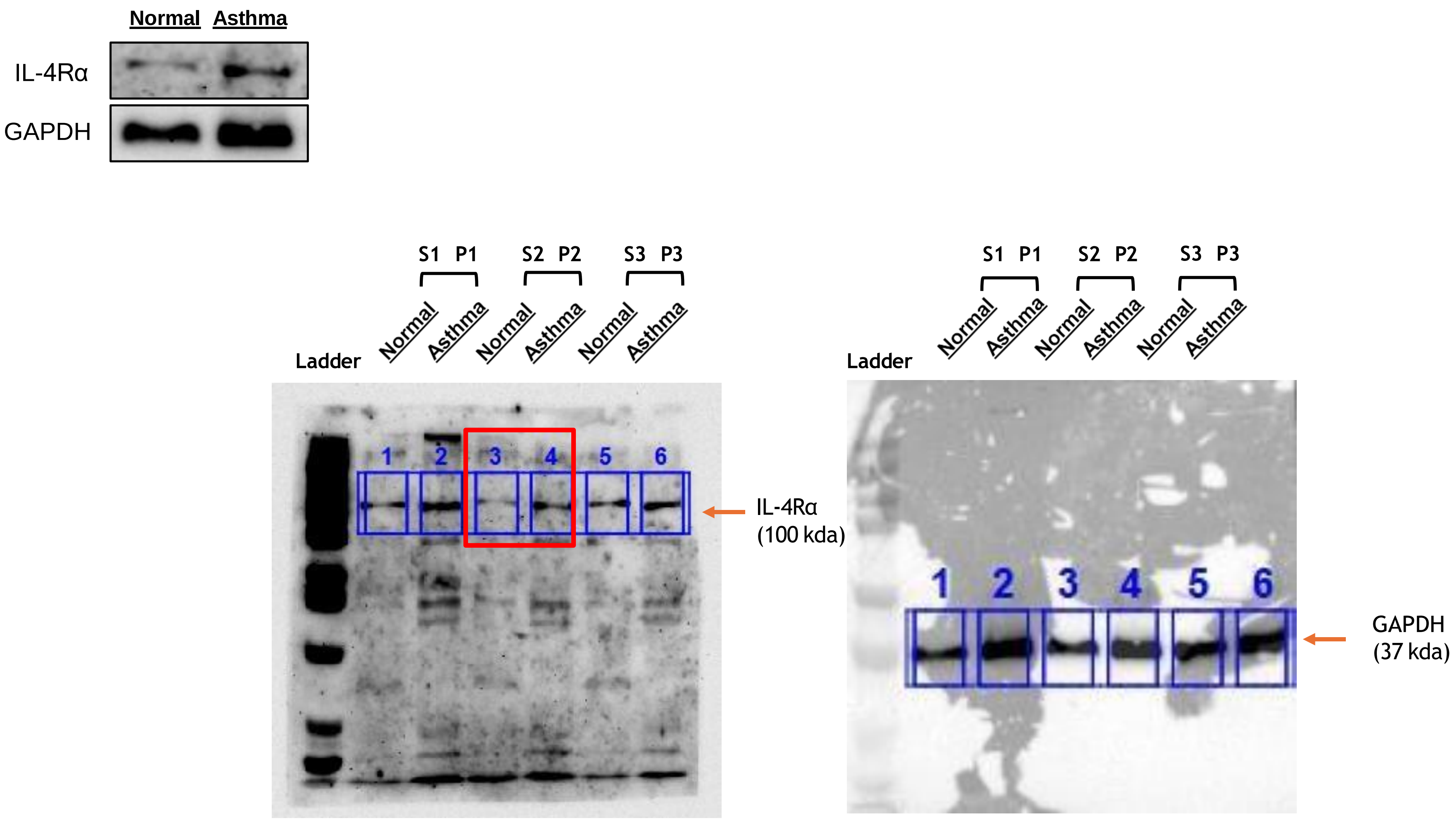

Figure 1D. Uncropped, full-length Western blot images for IL-4Rα blot upon rIL-4/rIL-13 stimulation featured in the main text, with molecular weight markers and protein band labels included from three different healthy subjects (S) and three different asthmatic patients (P).

Figure 1D

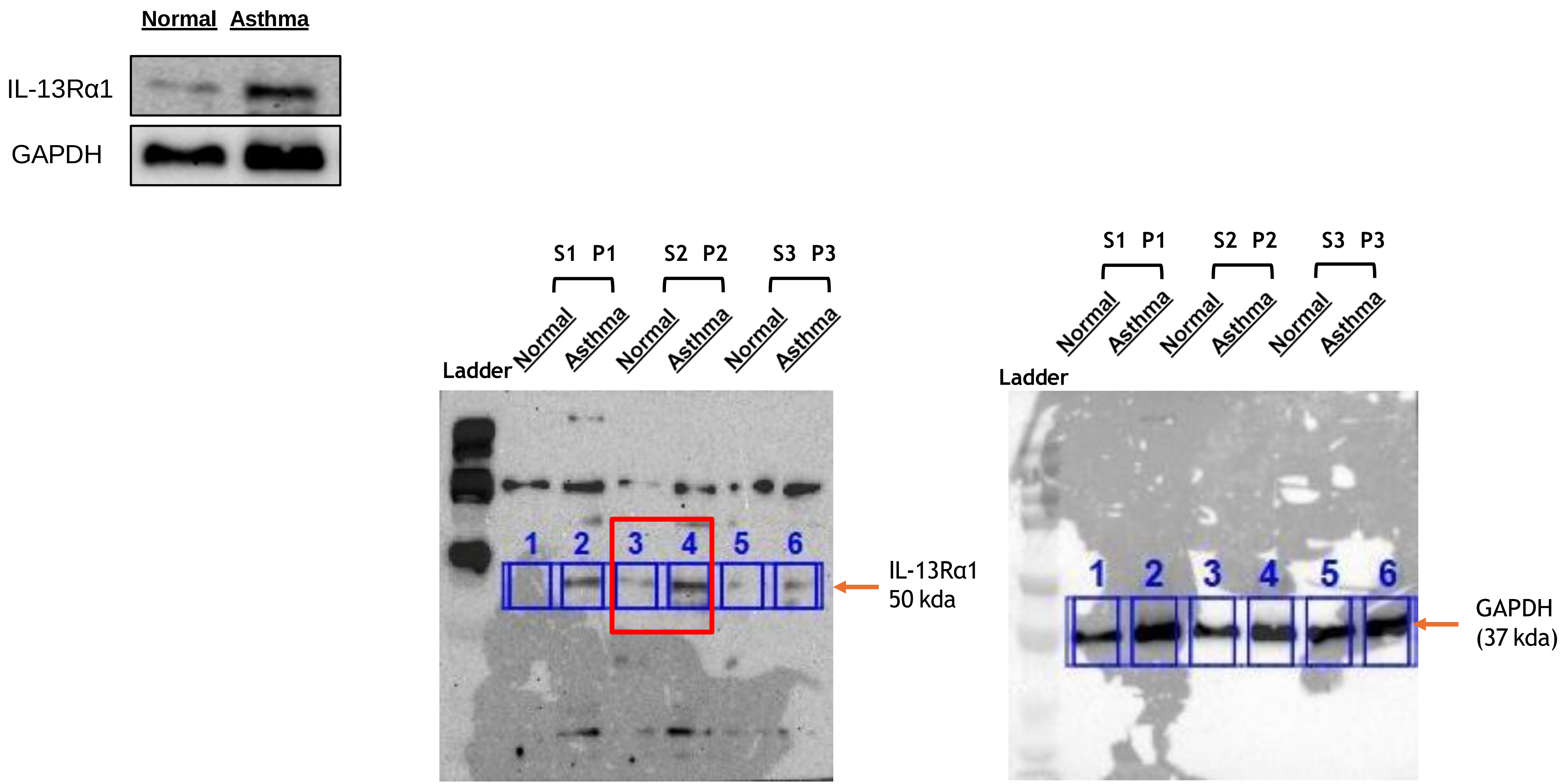

Figure 1D. Uncropped, full-length Western blot images for IL-13Rα1 blot upon rIL-4/rIL-13 stimulation featured in the main text, with molecular weight markers and protein band labels included from three different healthy subjects (S) and three different asthmatic patients (P).

Figure 2A

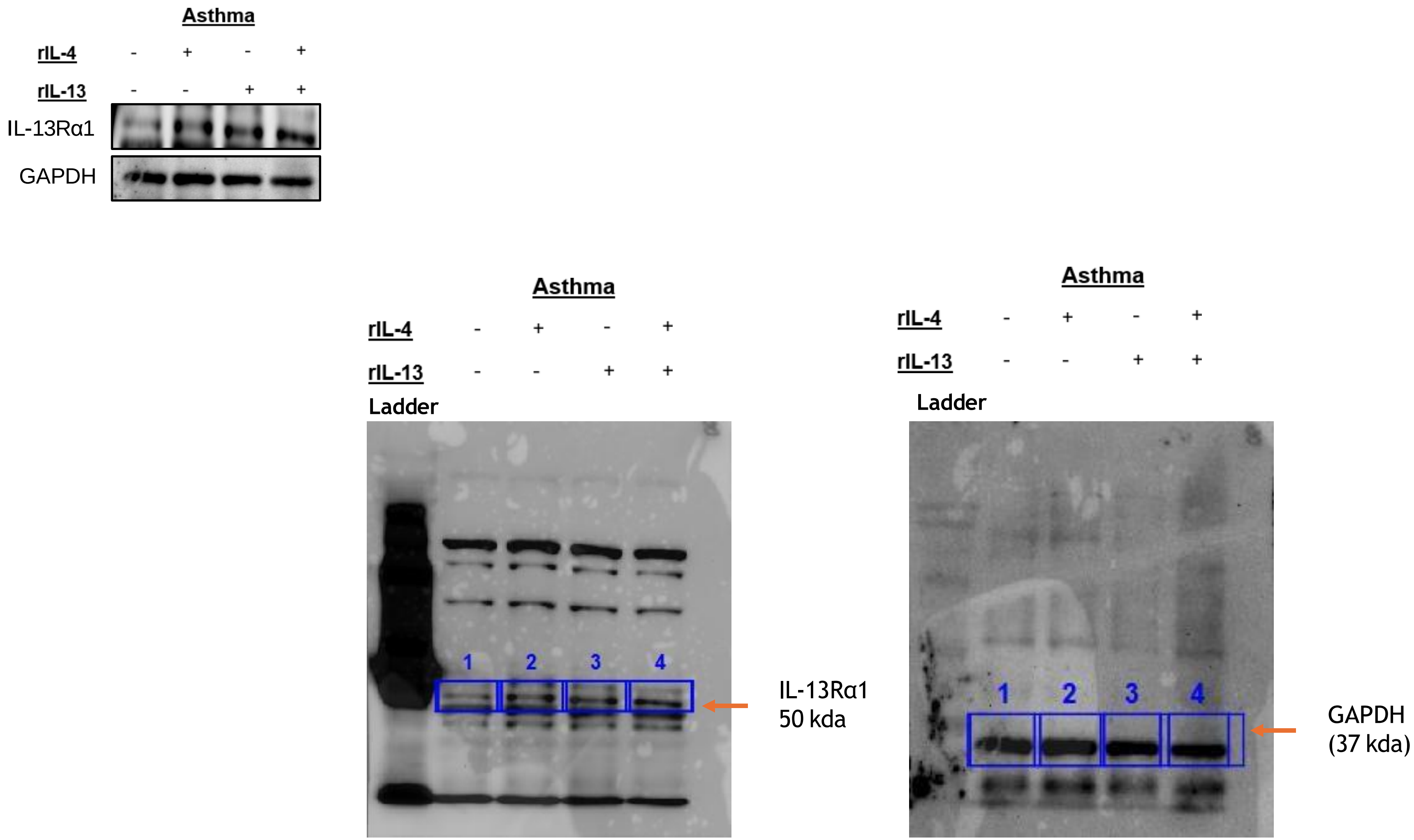

Figure 2A. Uncropped, full-length Western blot images for IL-13Rα1 blot upon rIL-4/rIL-13 stimulation featured in the main text, with molecular weight markers and protein band labels included.

Figure 2B

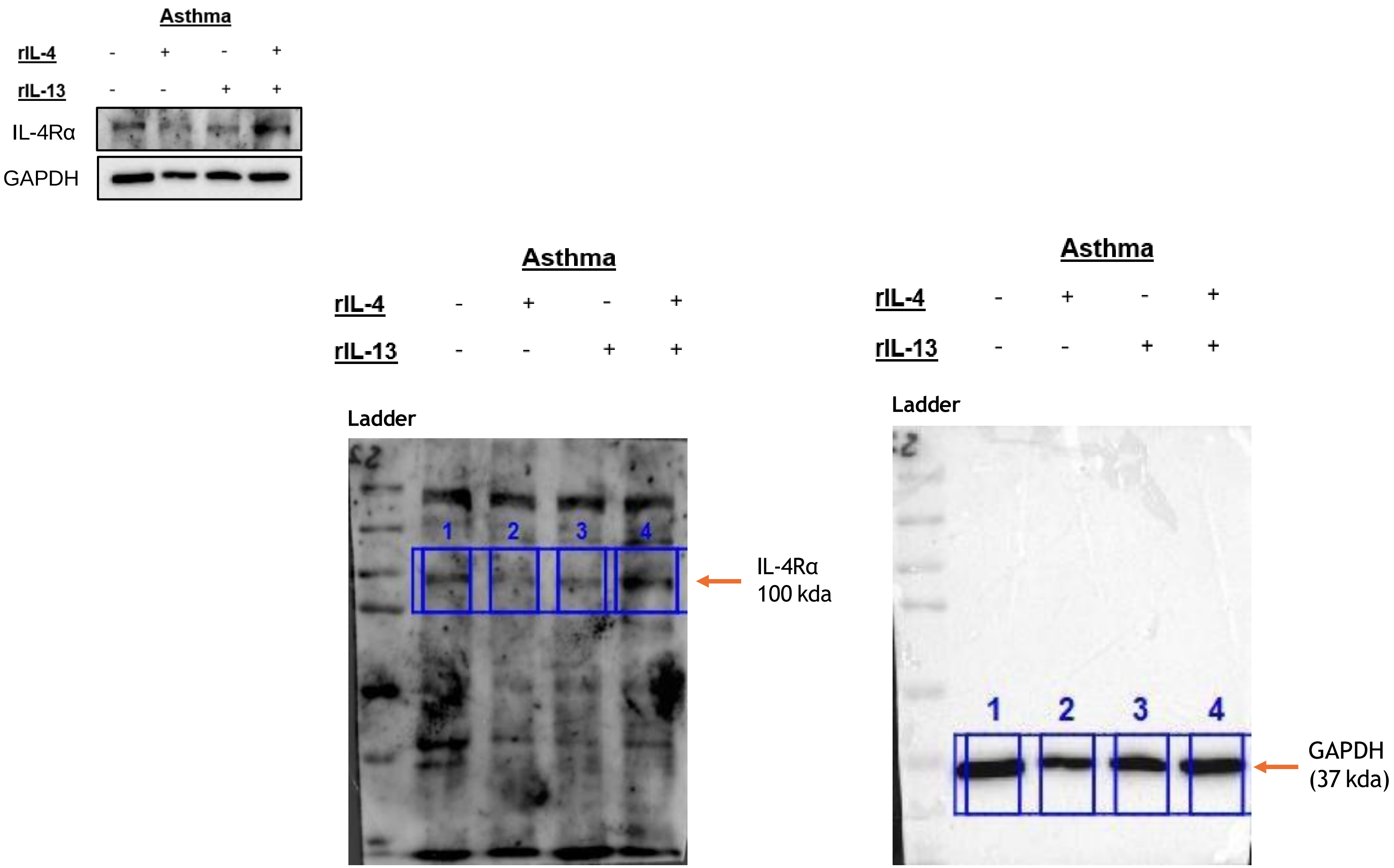

Figure 2B. Uncropped, full-length Western blot images for IL-4Rα blot upon rIL-4/rIL-13 stimulation featured in the main text, with molecular weight markers and protein band labels included.

Figure 2C

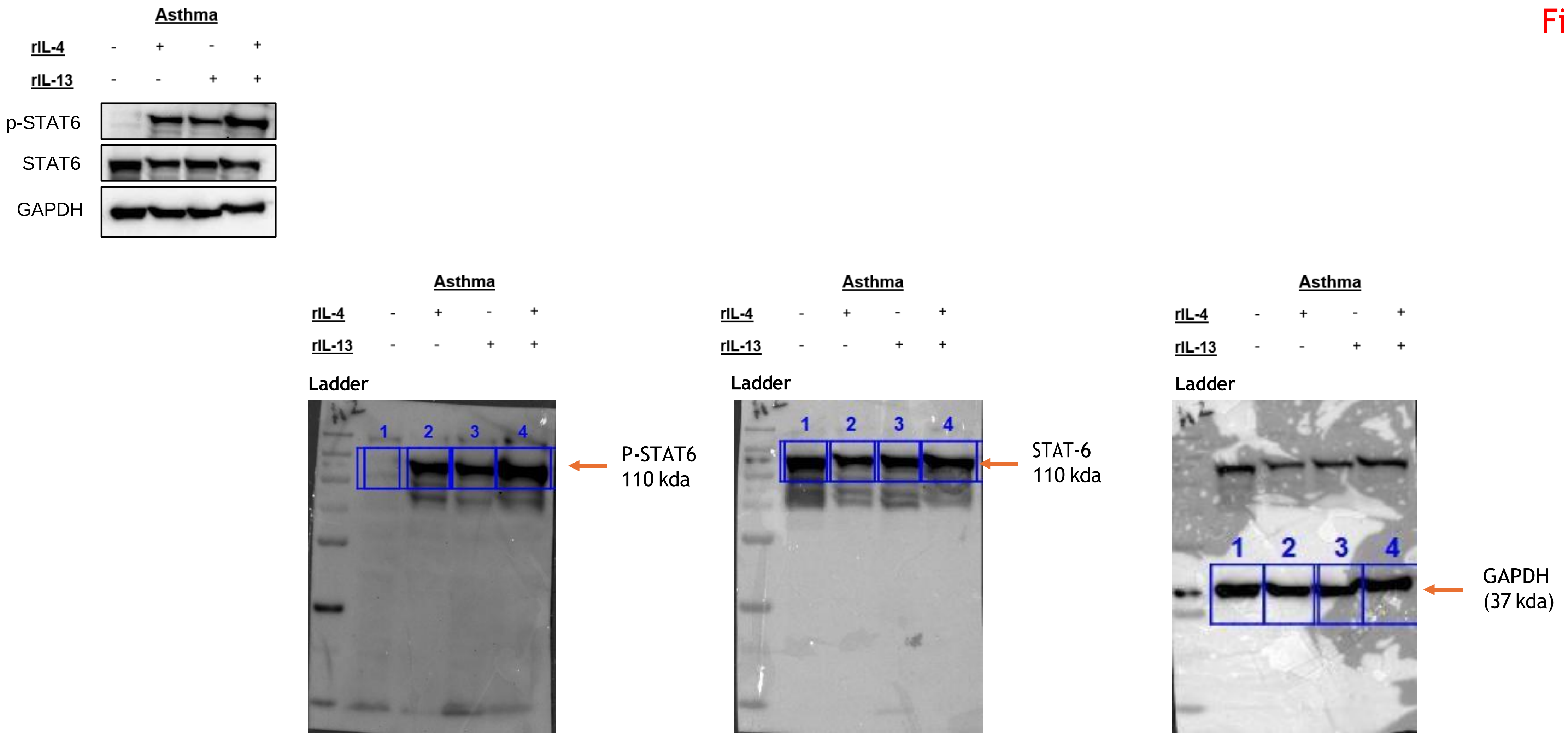

Figure 2C. Uncropped, full-length Western blot images for STAT6 phosphorylation blots upon rIL-4/rIL-13 stimulation featured in the main text, with molecular weight markers and protein band labels included.

Figure 3A

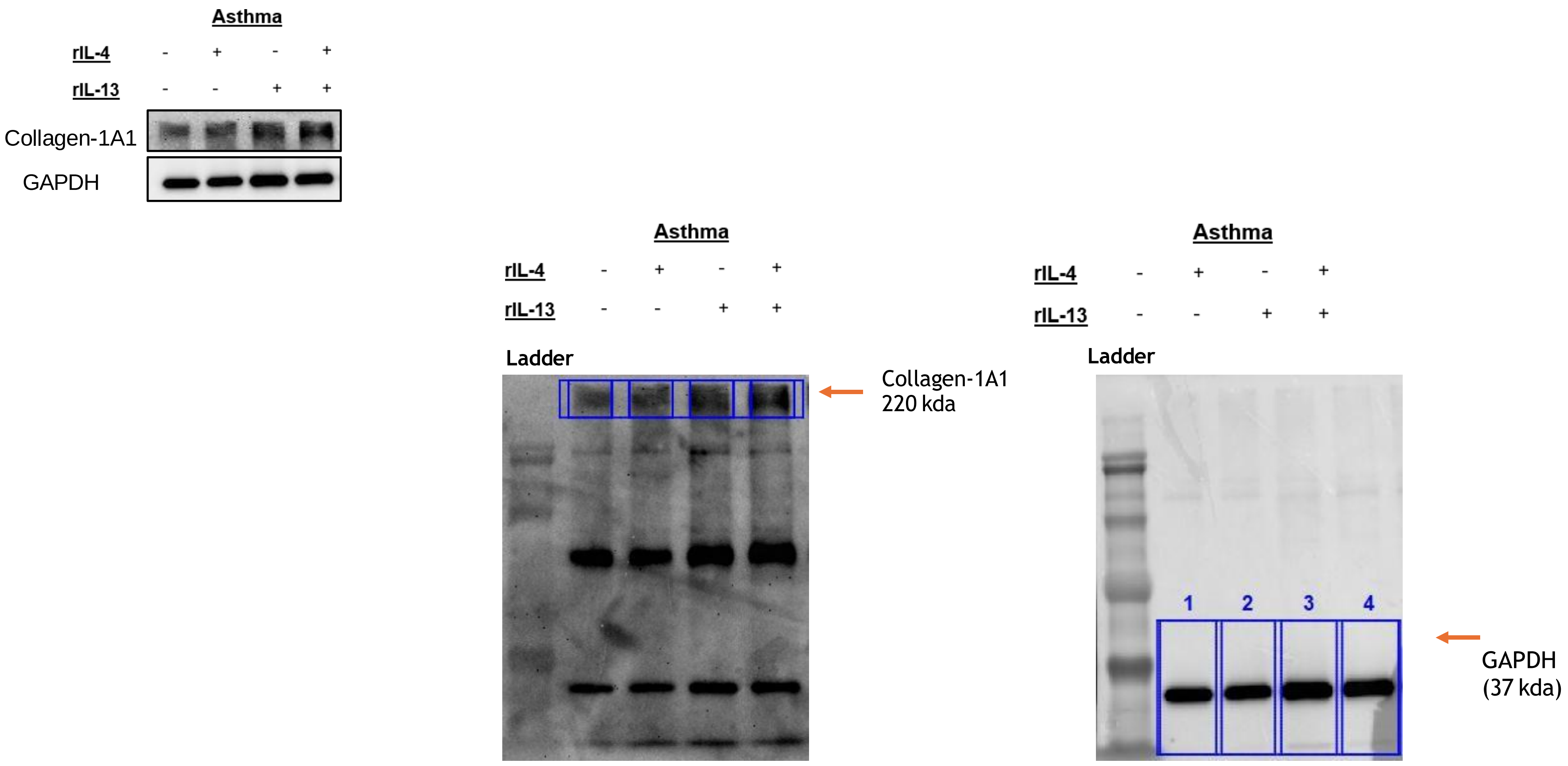

Figure 3A. Uncropped, full-length Western blot images for collagen-1A1 blot upon rIL-4/rIL-13 stimulation featured in the main text, with molecular weight markers and protein band labels included.

Figure 3A

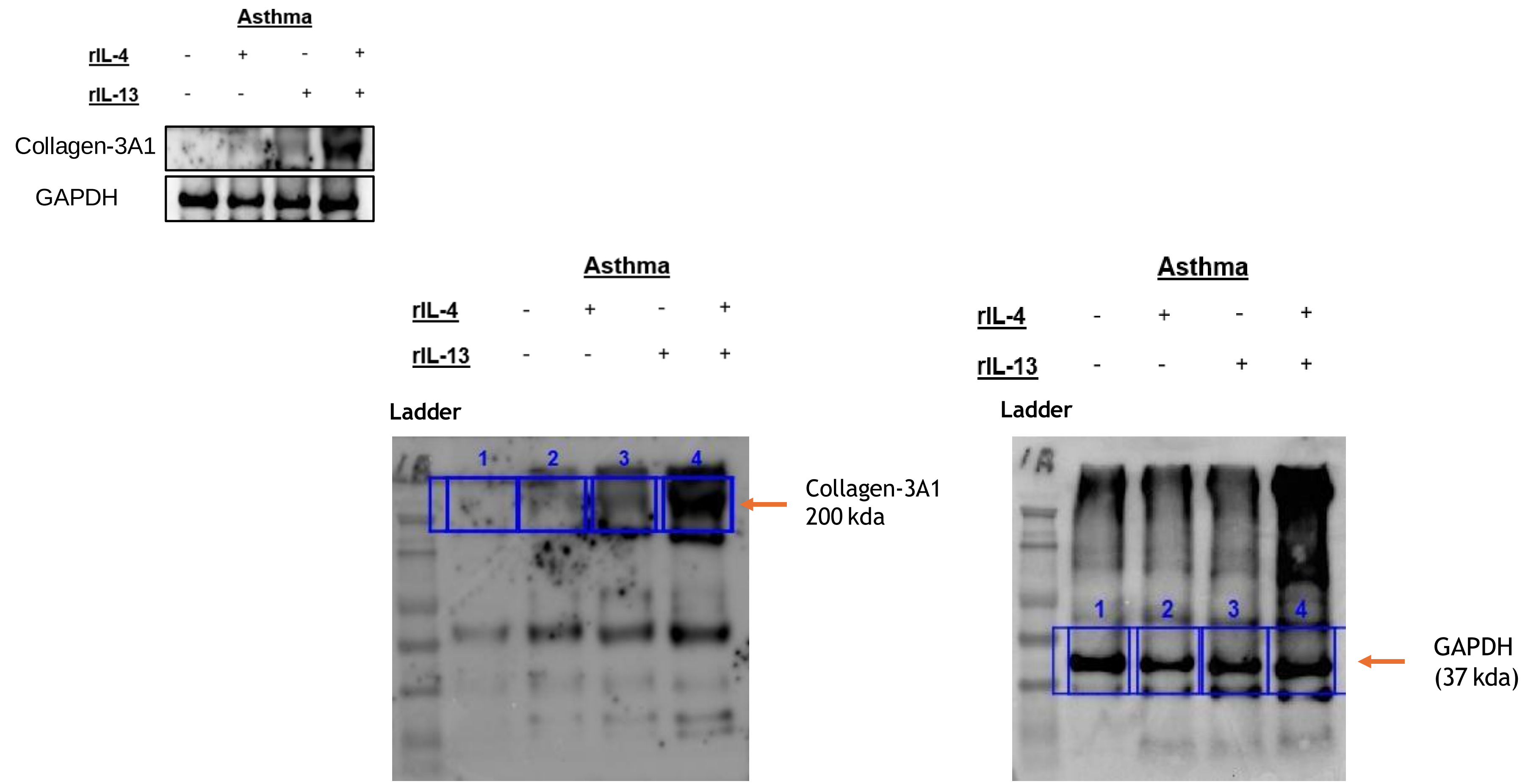

Figure 3A. Uncropped, full-length Western blot images for collagen-3A1 blot upon rIL-4/rIL-13 stimulation featured in the main text, with molecular weight markers and protein band labels included.

Figure 3A

Asthma

rIL-4	-	+	-	+
rIL-13	-	-	+	+

Fibronectin

GAPDH

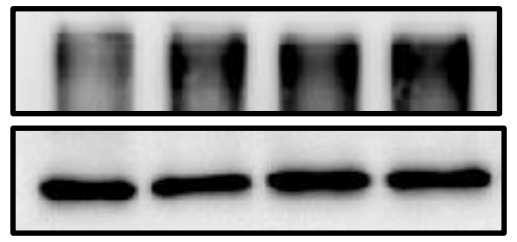

Asthma

rIL-4	-	+	-	+
rIL-13	-	-	+	+

Ladder

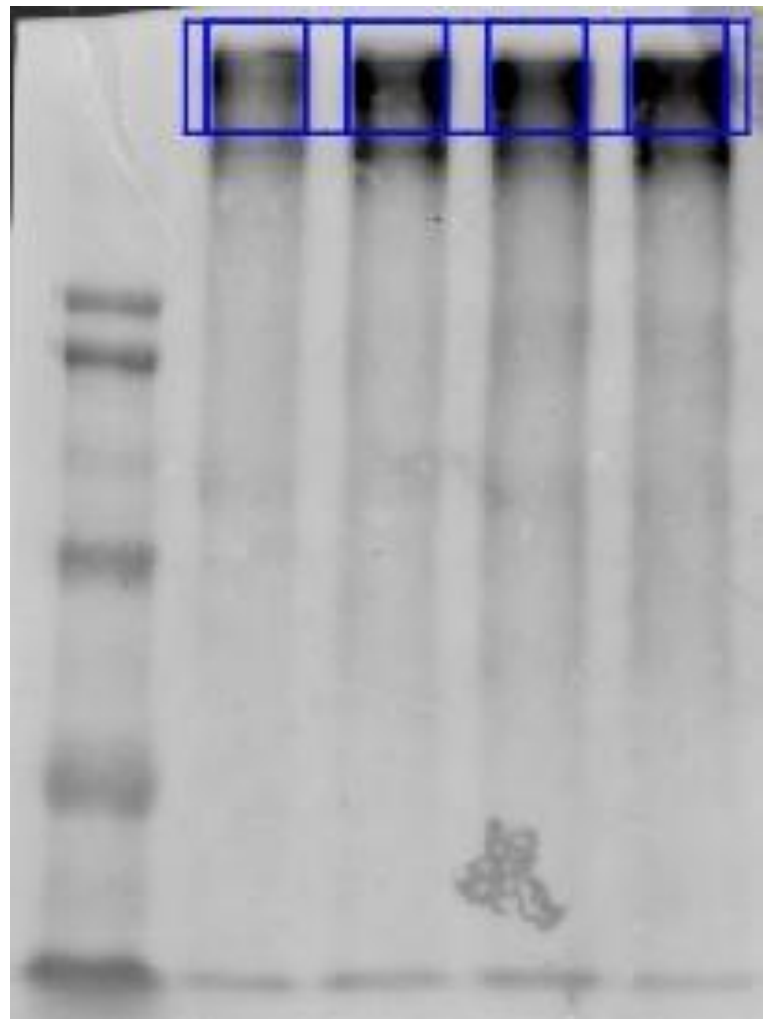

Fibronectin
300 kda

Asthma

rIL-4	-	+	-	+
rIL-13	-	-	+	+

Ladder

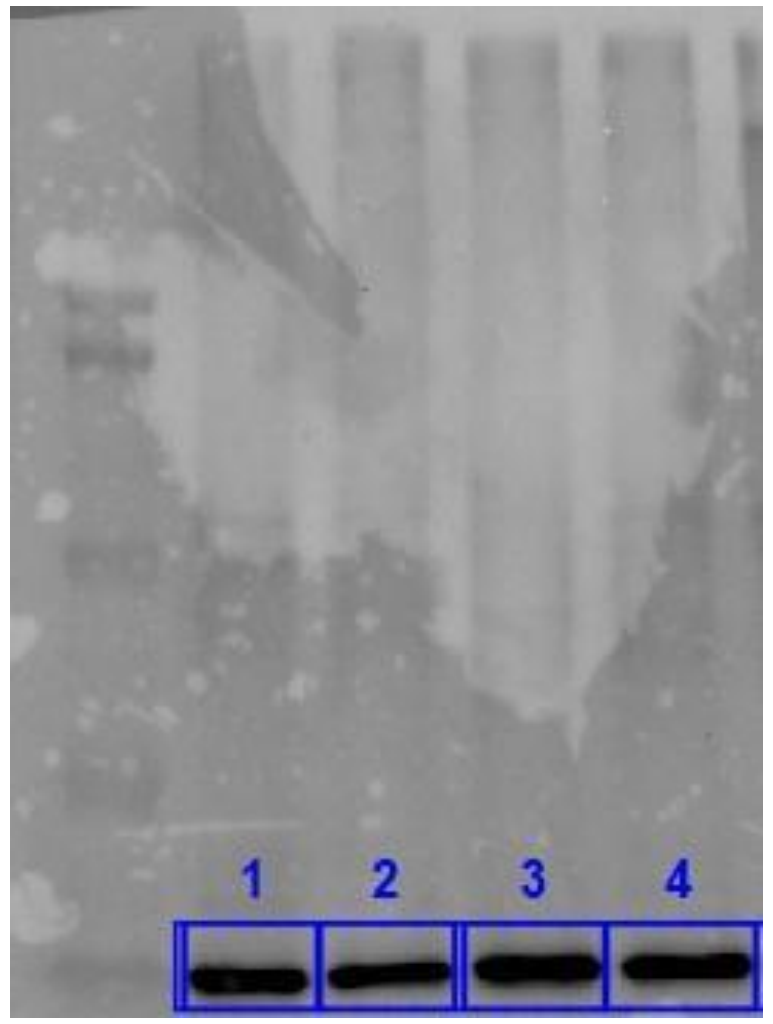

GAPDH
(37 kda)

Figure 3A. Uncropped, full-length Western blot images for fibronectin blot upon rIL-4/rIL-13 stimulation featured in the main text, with molecular weight markers and protein band labels included.

Figure 3A

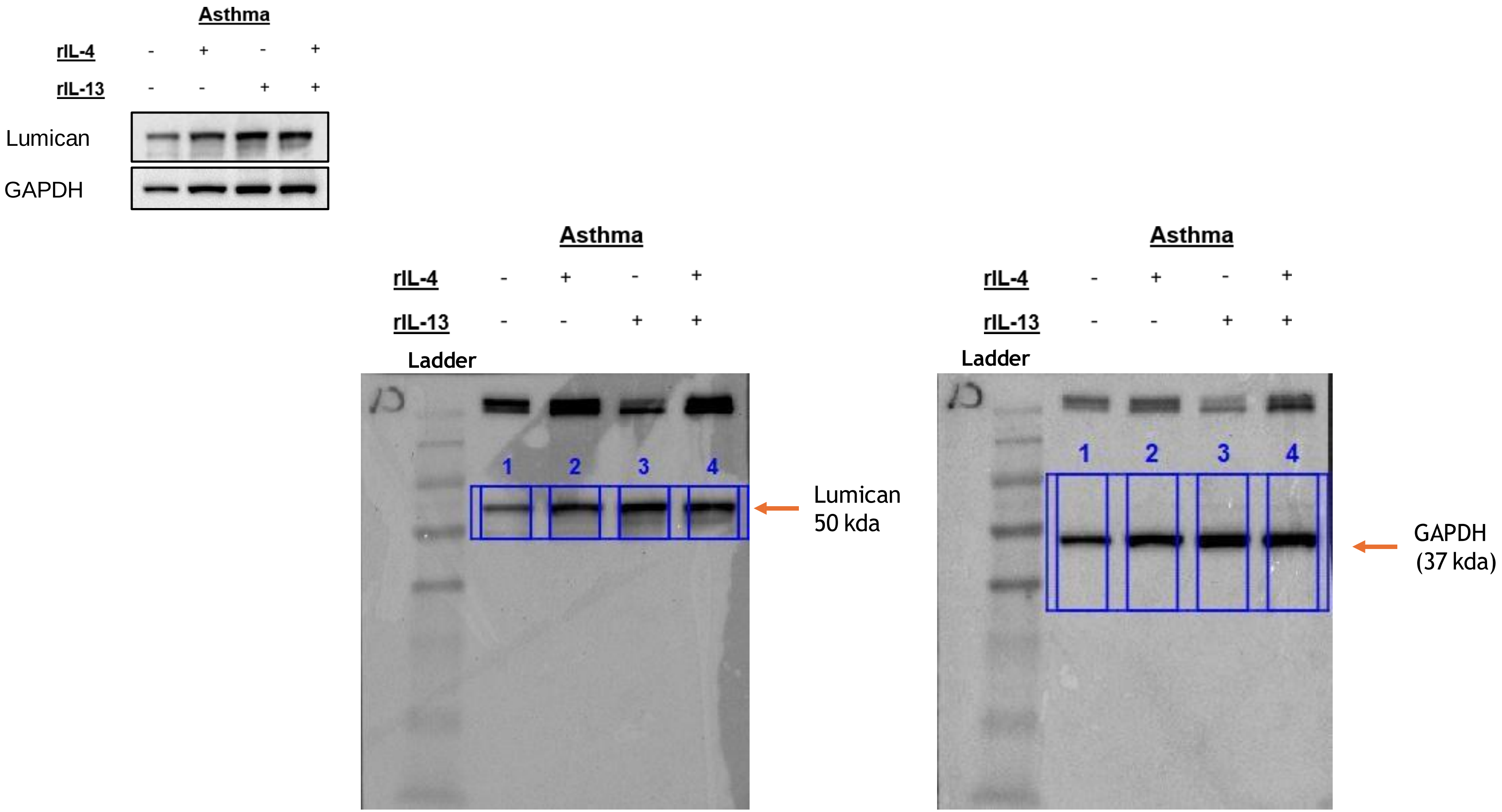

Figure 3A. Uncropped, full-length Western blot images for lumican blot upon rIL-4/rIL-13 stimulation featured in the main text, with molecular weight markers and protein band labels included.

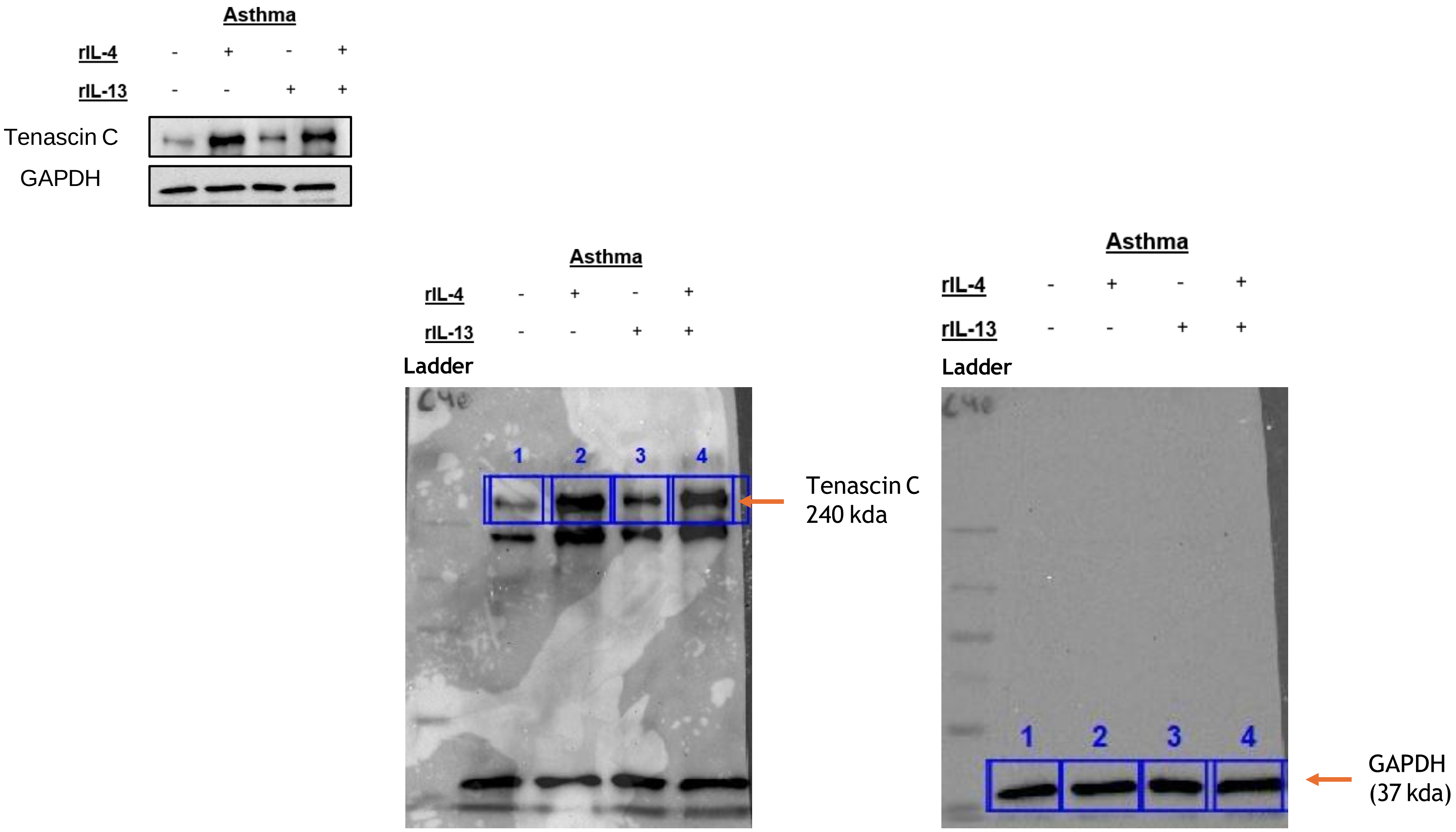

Figure 3A

Figure 3A. Uncropped, full-length Western blot images for tenascin C blot upon rIL-4/rIL-13 stimulation featured in the main text, with molecular weight markers and protein band labels included.

Figure 3B

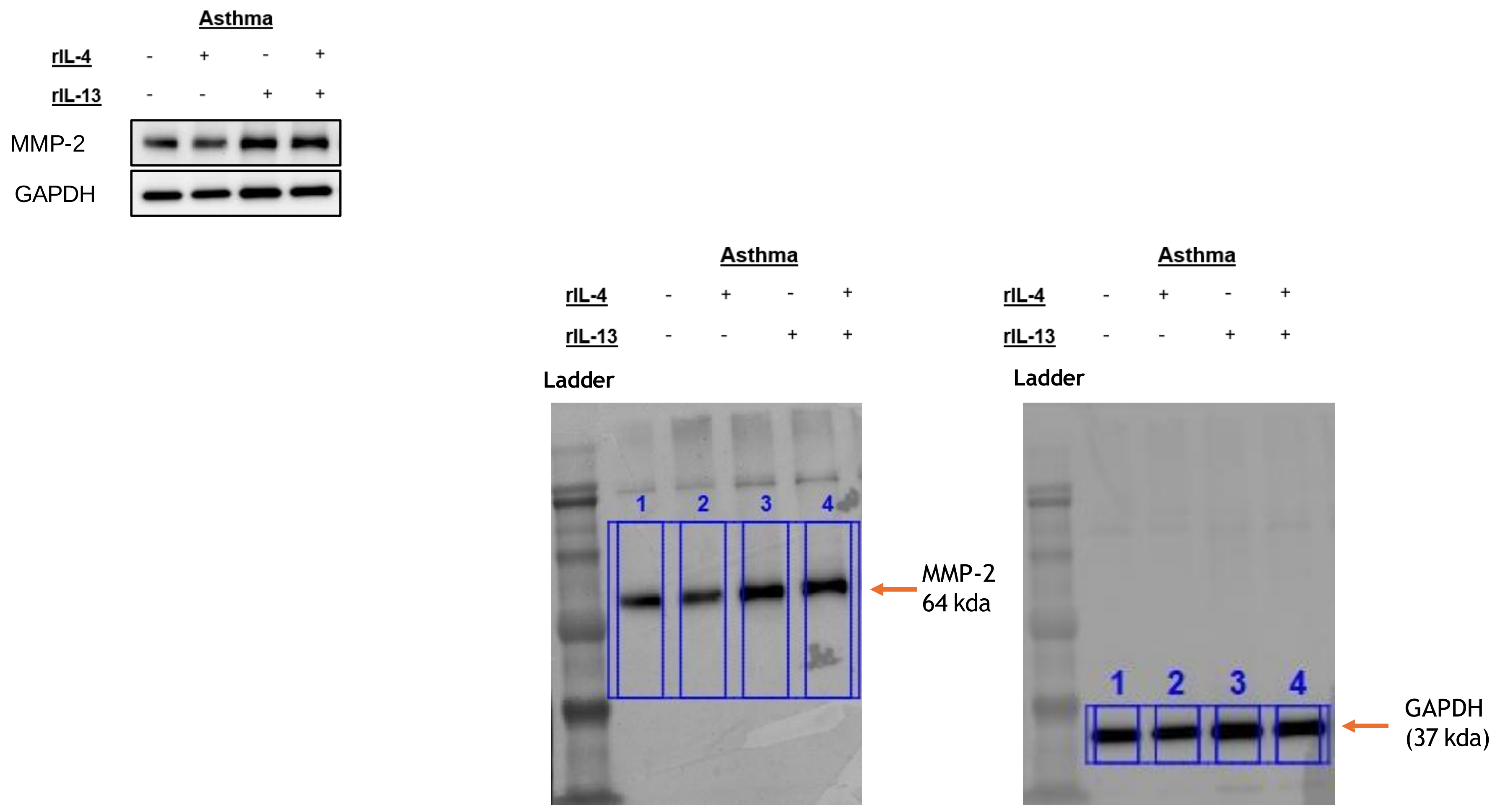

Figure 3B. Uncropped, full-length Western blot images for MMP-2 blot upon rIL-4/rIL-13 stimulation featured in the main text, with molecular weight markers and protein band labels included.

Figure 3B

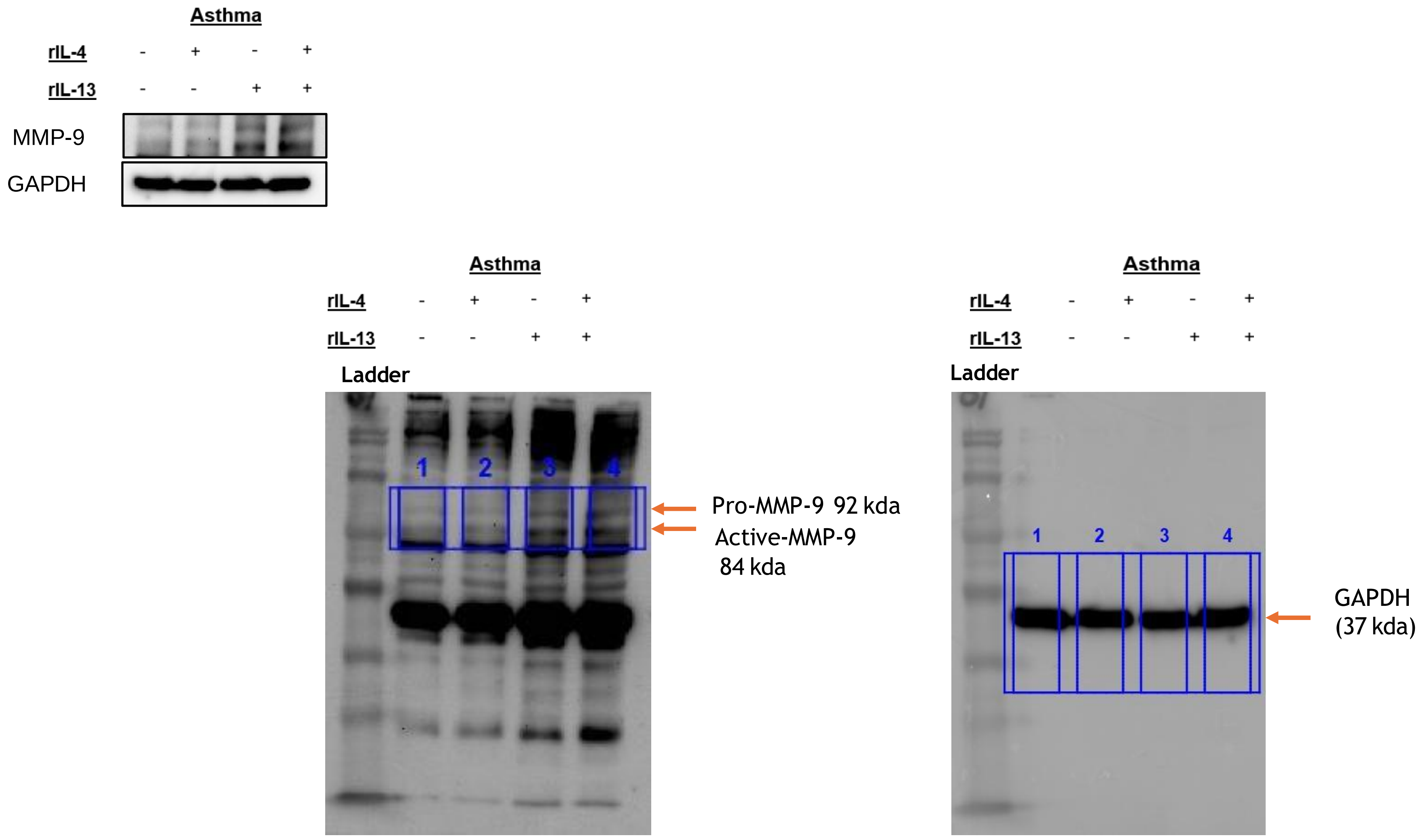

Figure 3B. Uncropped, full-length Western blot images for MMP-9 blot upon rIL-4/rIL-13 stimulation featured in the main text, with molecular weight markers and protein band labels included.

Figure 3B

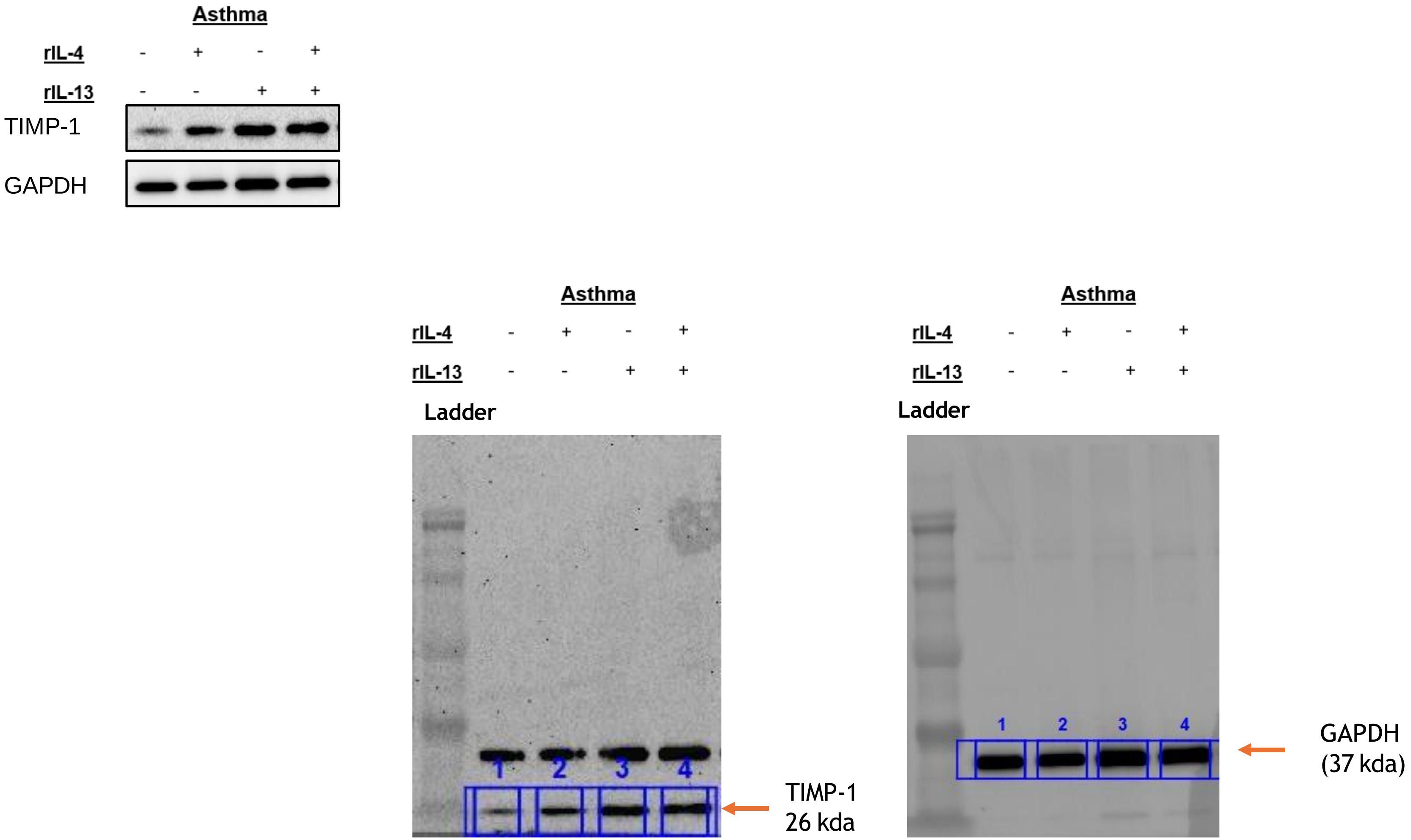

Figure 3B. Uncropped, full-length Western blot images for TIMP-1 blot upon rIL-4/rIL-13 stimulation featured in the main text, with molecular weight markers and protein band labels included.

Figure 3B

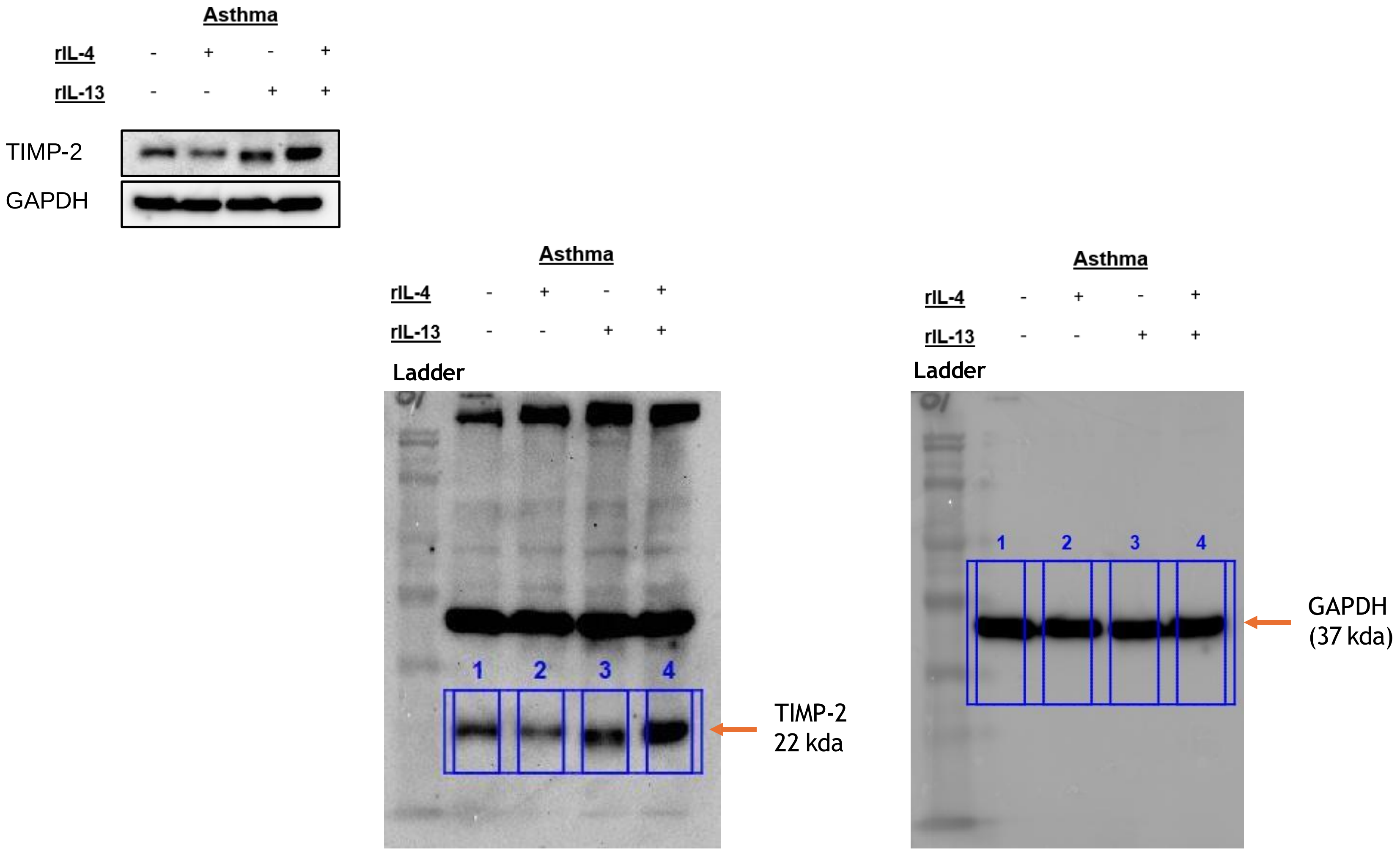

Figure 3B. Uncropped, full-length Western blot images for TIMP-2 blot upon rIL-4/rIL-13 stimulation featured in the main text, with molecular weight markers and protein band labels included.

Figure 4A

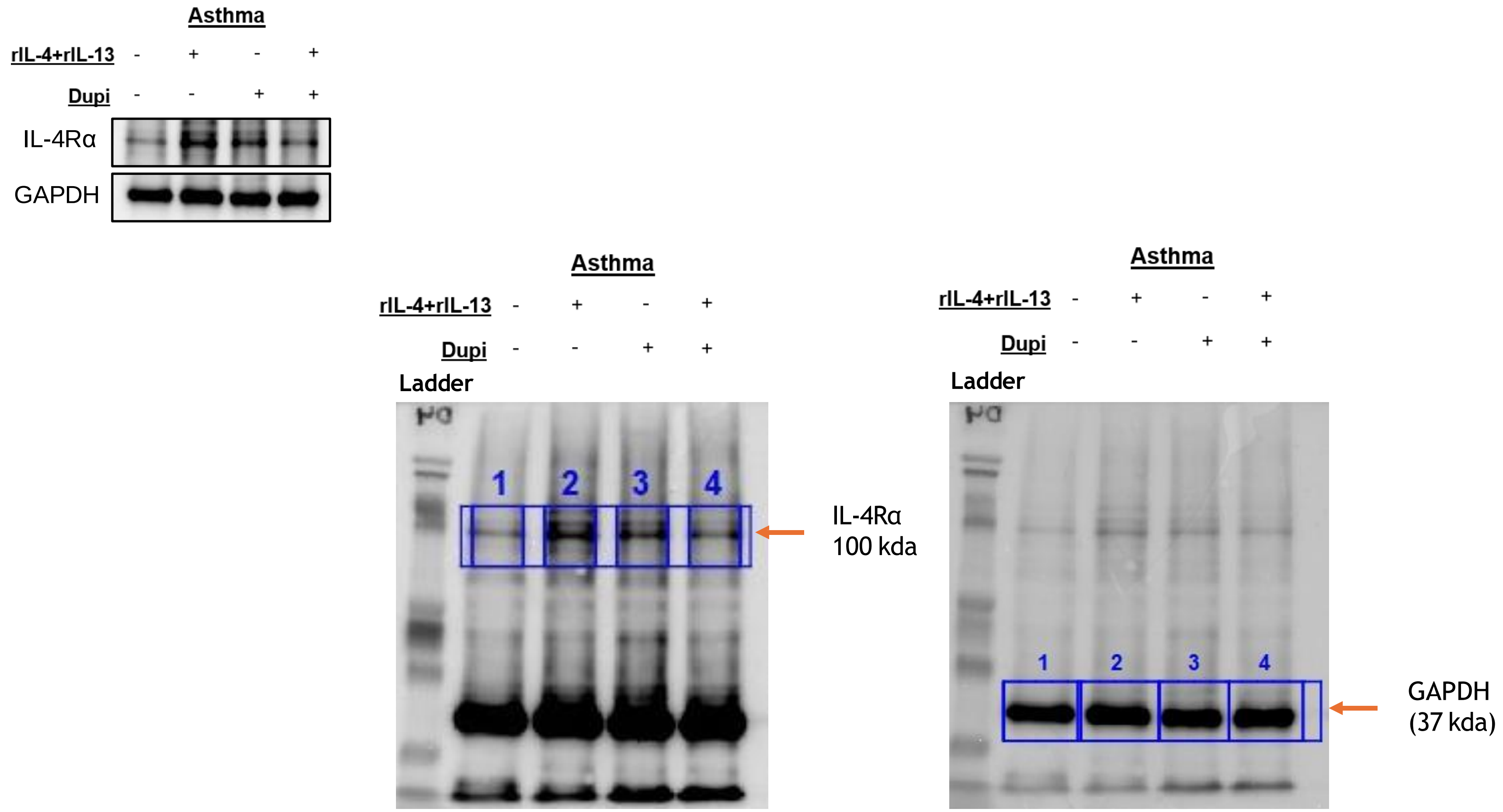

Figure 4A. Uncropped, full-length Western blot images for IL-4Rα blot upon Dupi treatment featured in the main text, with molecular weight markers and protein band labels included.

Figure 4B

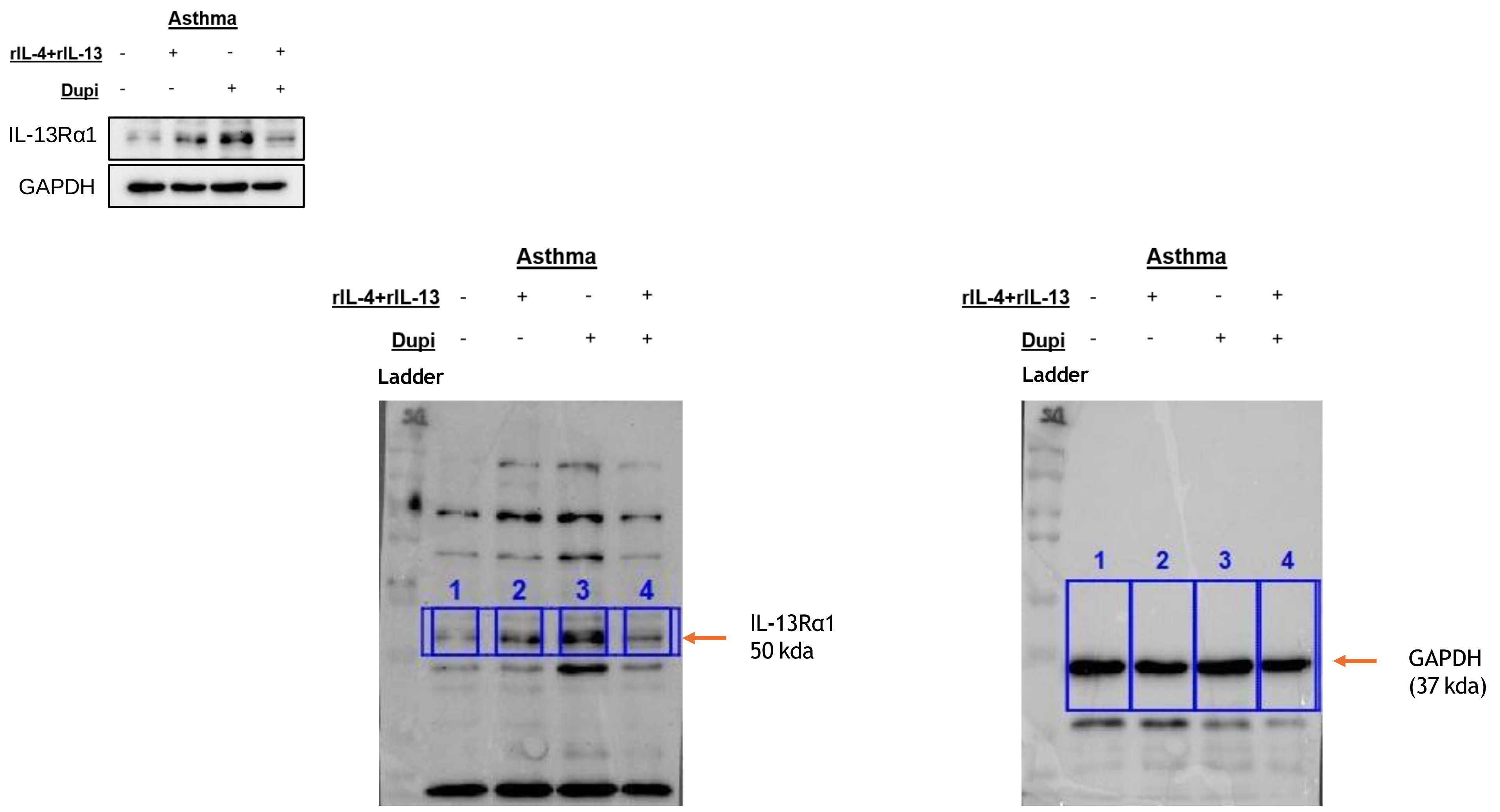

Figure 4B. Uncropped, full-length Western blot images for IL-13Rα1 blot upon Dupi treatment featured in the main text, with molecular weight markers and protein band labels included.

Figure 4C

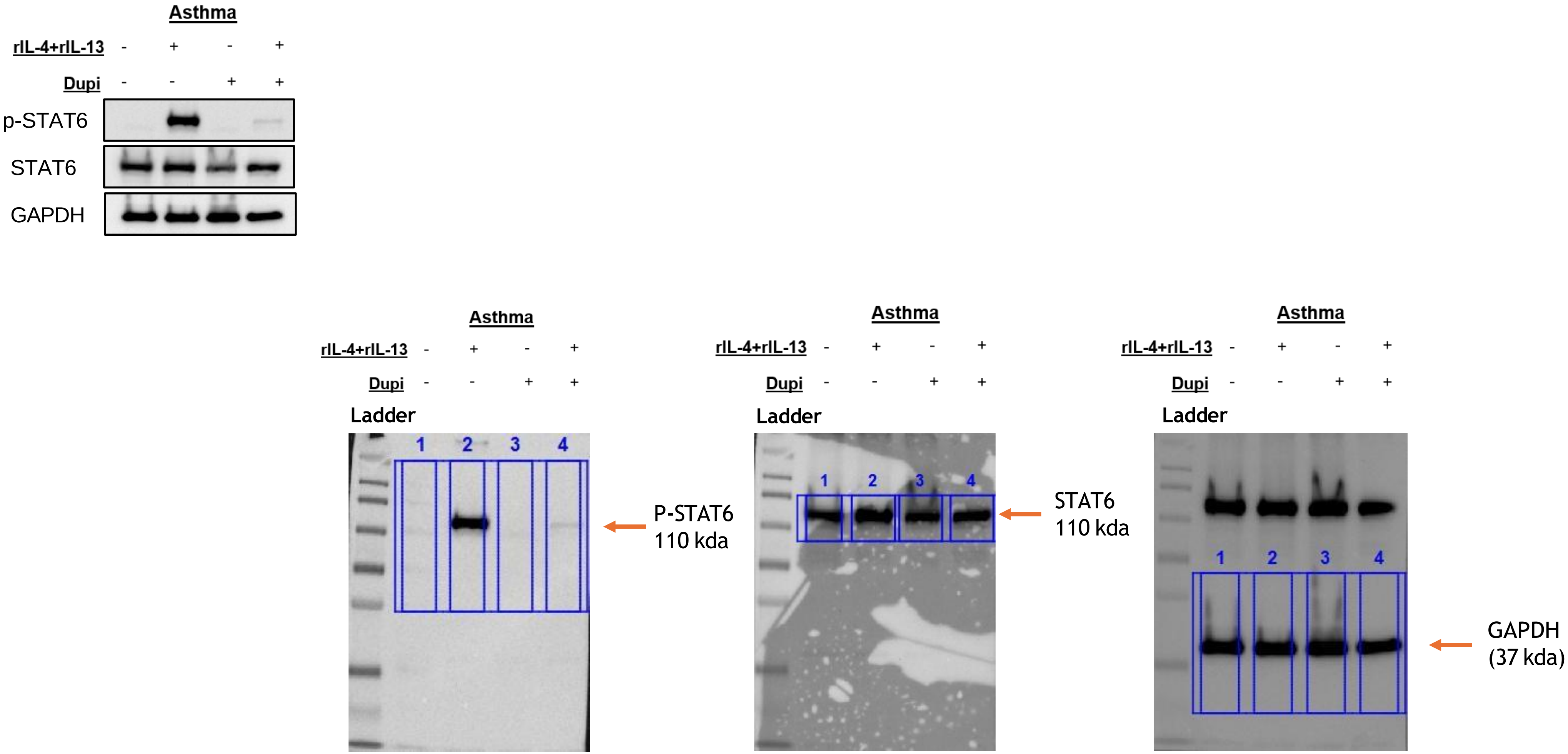

Figure 4C. Uncropped, full-length Western blot images for STAT6 phosphorylation blots upon Dupi treatment featured in the main text, with molecular weight markers and protein band labels included.

Figure 5A

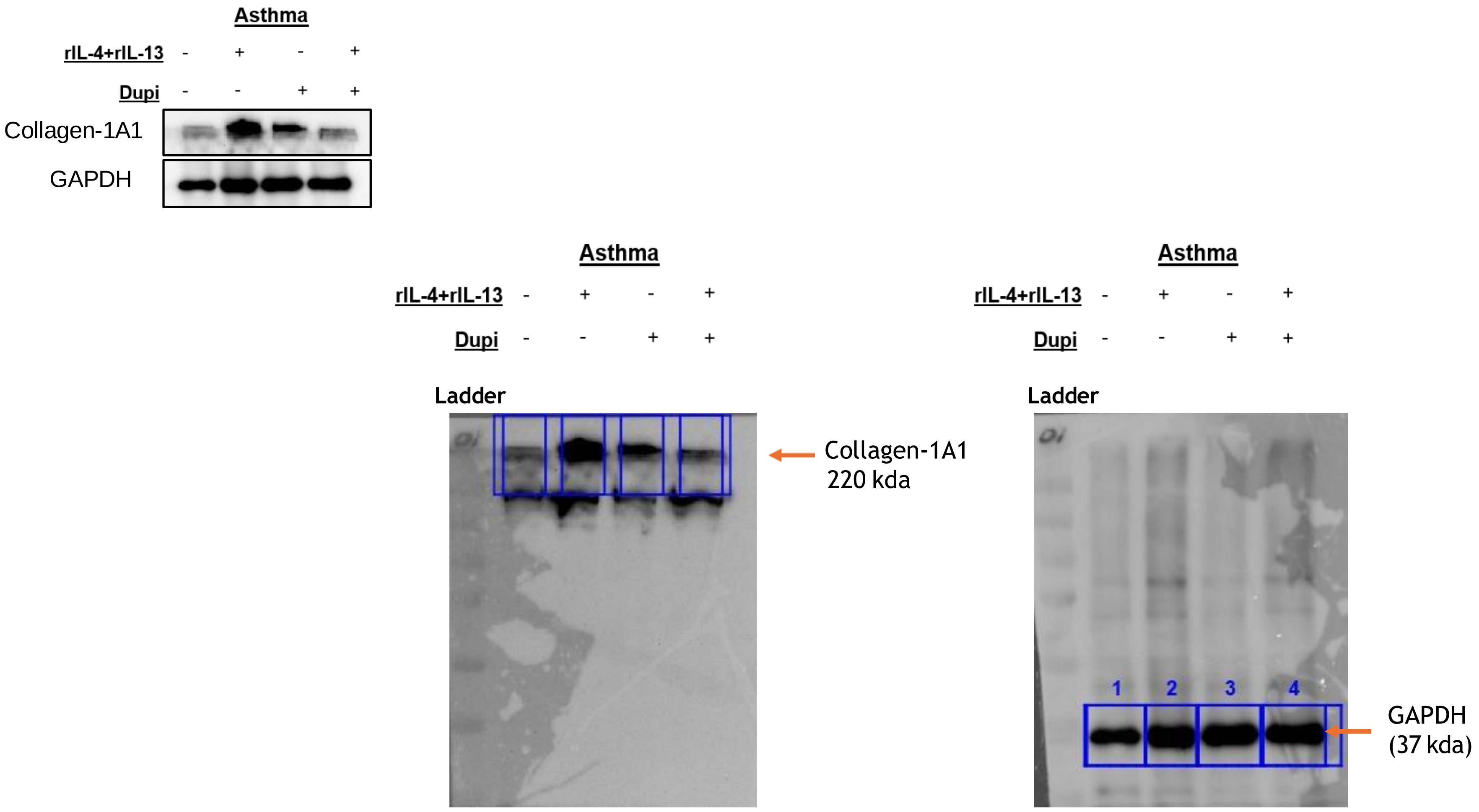

Figure 5A. Uncropped, full-length Western blot images for collagen-1A1 blot upon Dupi treatment featured in the main text, with molecular weight markers and protein band labels included.

Figure 5A

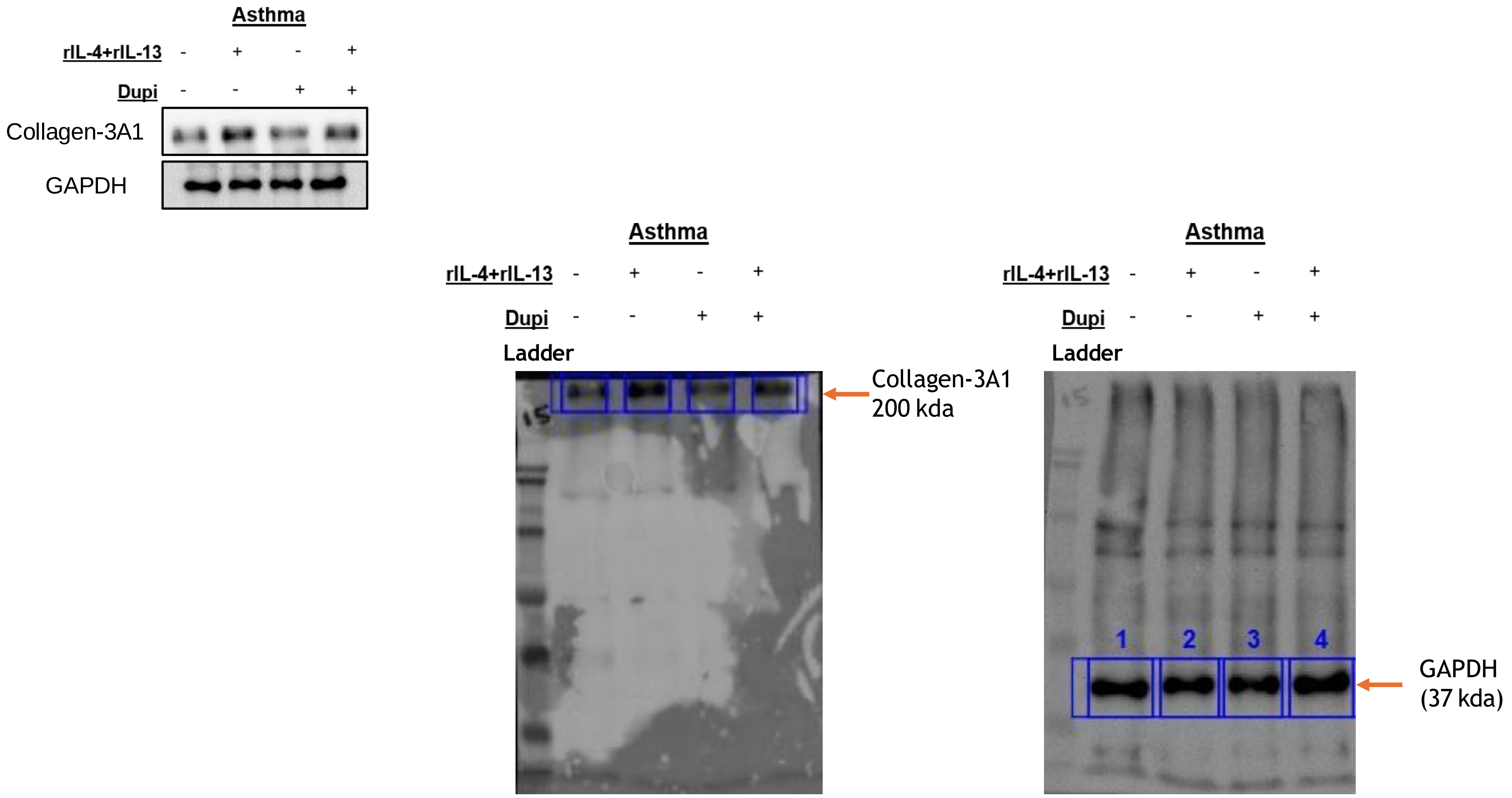

Figure 5A. Uncropped, full-length Western blot images for collagen-3A1 blot upon Dupi treatment featured in the main text, with molecular weight markers and protein band labels included.

Figure 5A

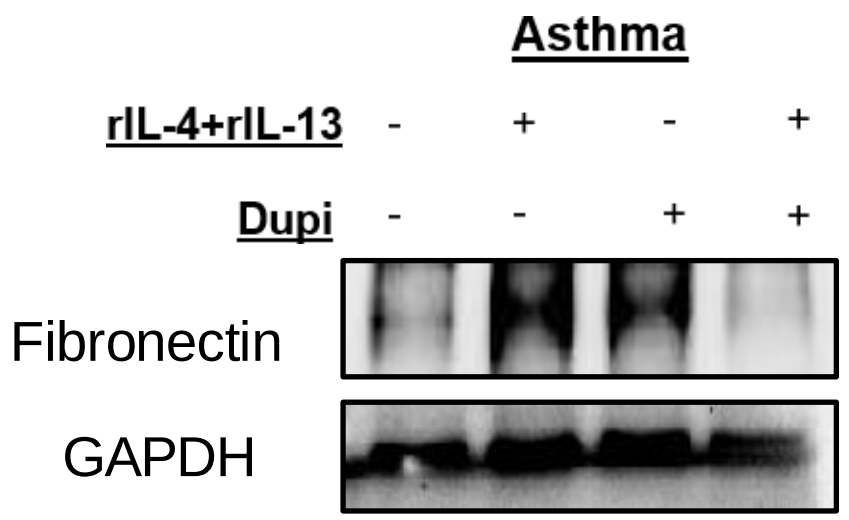

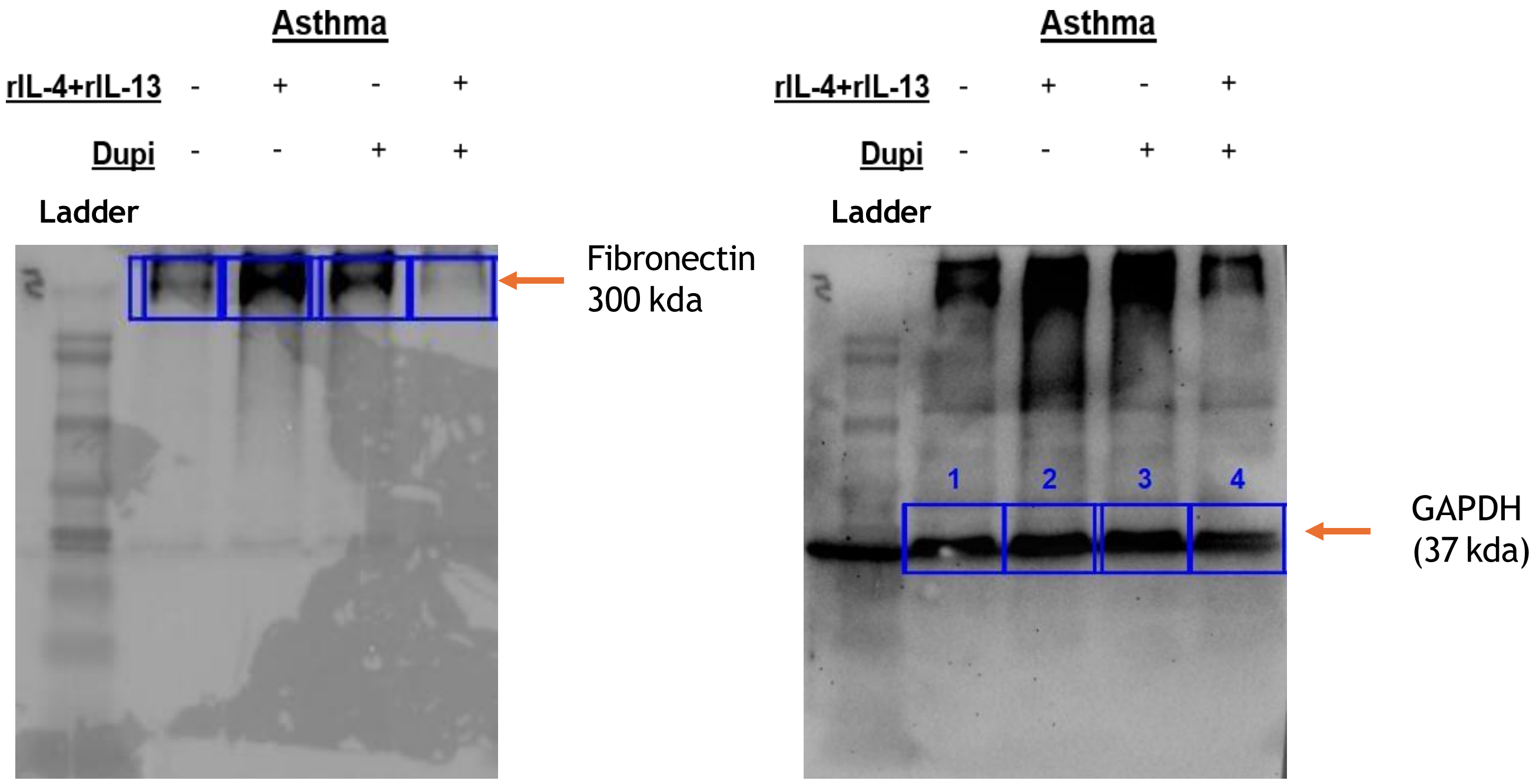

Figure 5A. Uncropped, full-length Western blot images for fibronectin blot upon Dupi treatment featured in the main text, with molecular weight markers and protein band labels included.

Figure 5A

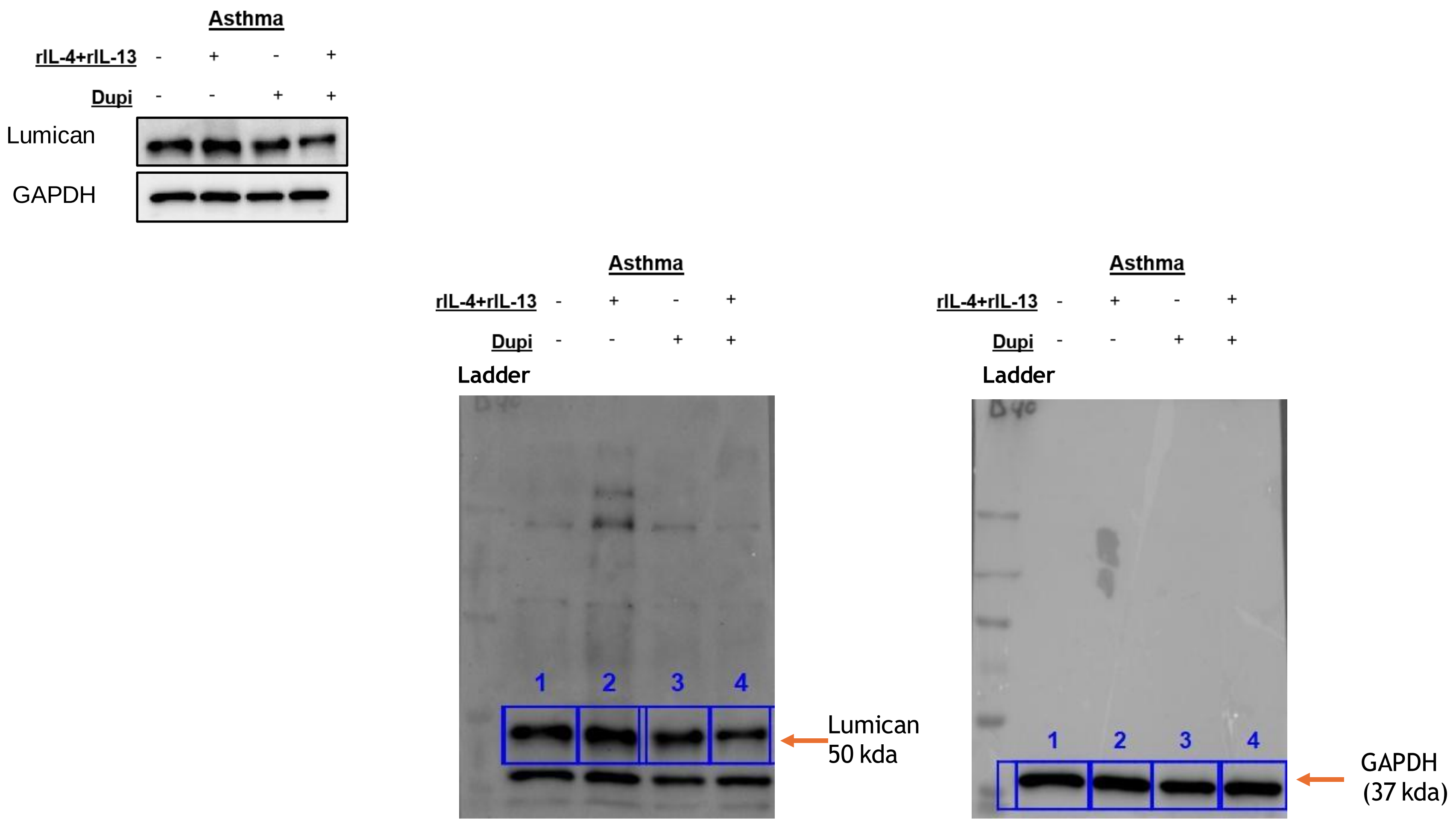

Figure 5A. Uncropped, full-length Western blot images for lumican blot upon Dupi treatment featured in the main text, with molecular weight markers and protein band labels included.

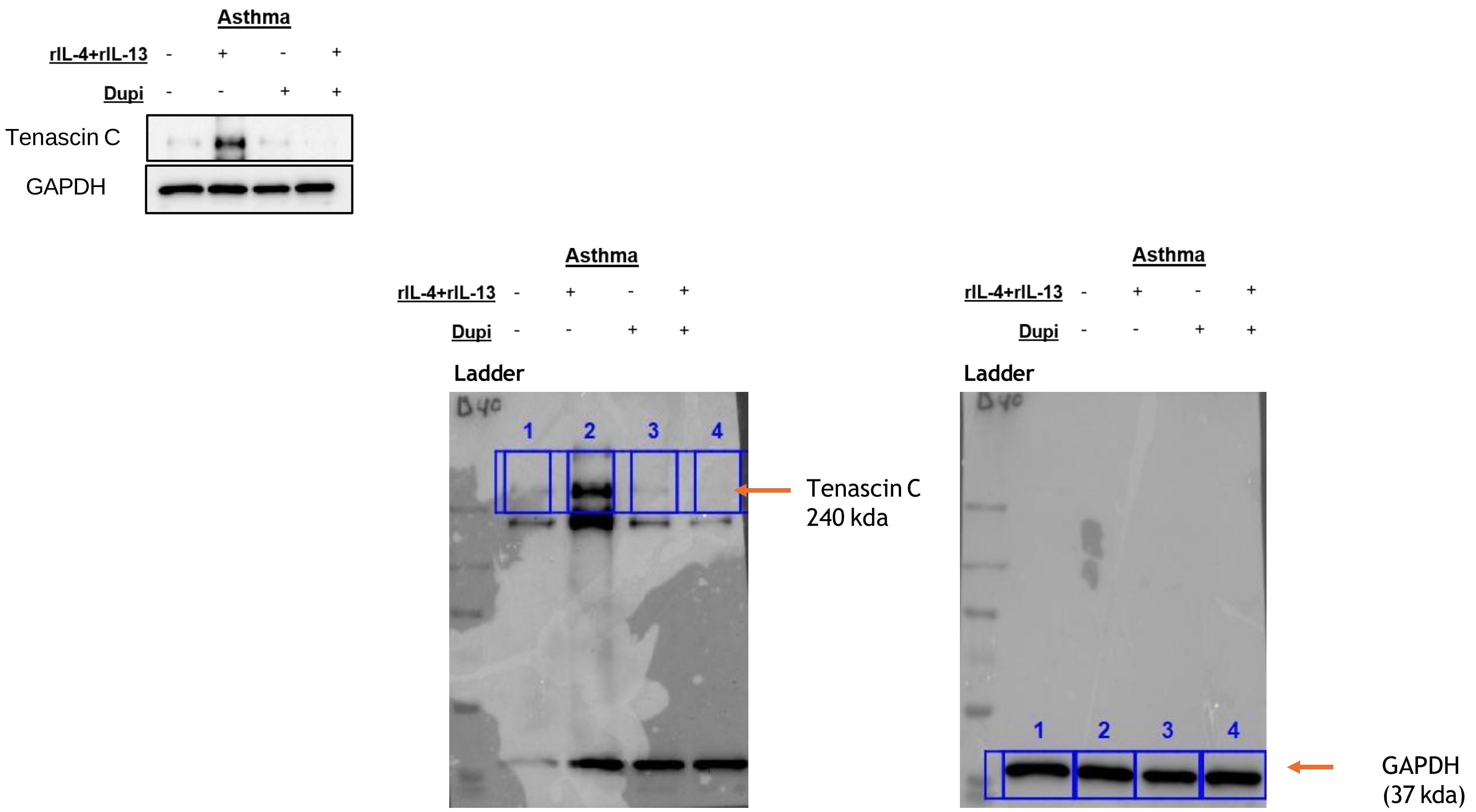

Figure 5A. Uncropped, full-length Western blot images for tenascin C blot upon Dupi treatment featured in the main text, with molecular weight markers and protein band labels included.

Figure 5B

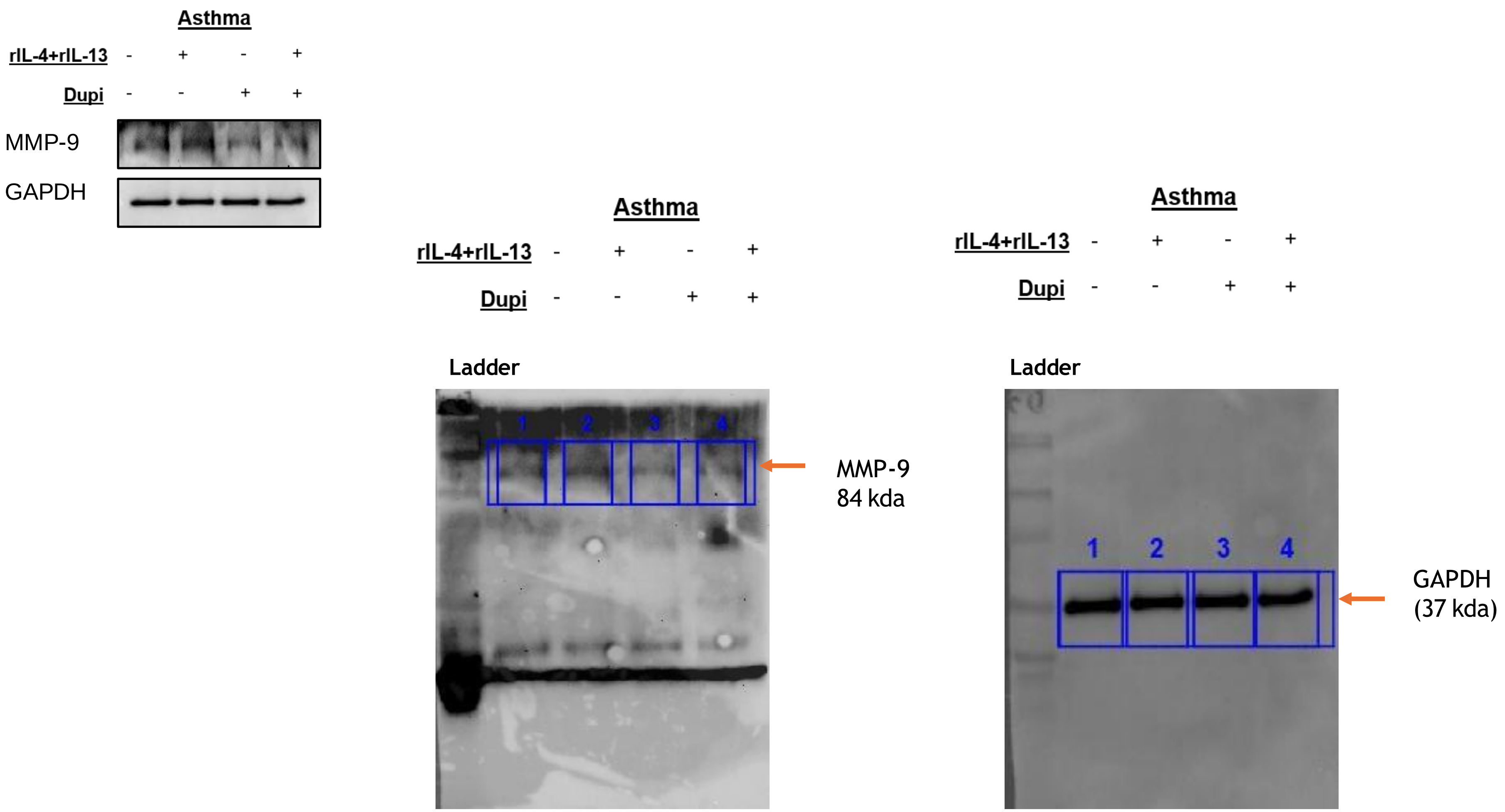

Figure 5B. Uncropped, full-length Western blot images for MMP-9 blot upon Dupi treatment featured in the main text, with molecular weight markers and protein band labels included.

Figure 5B

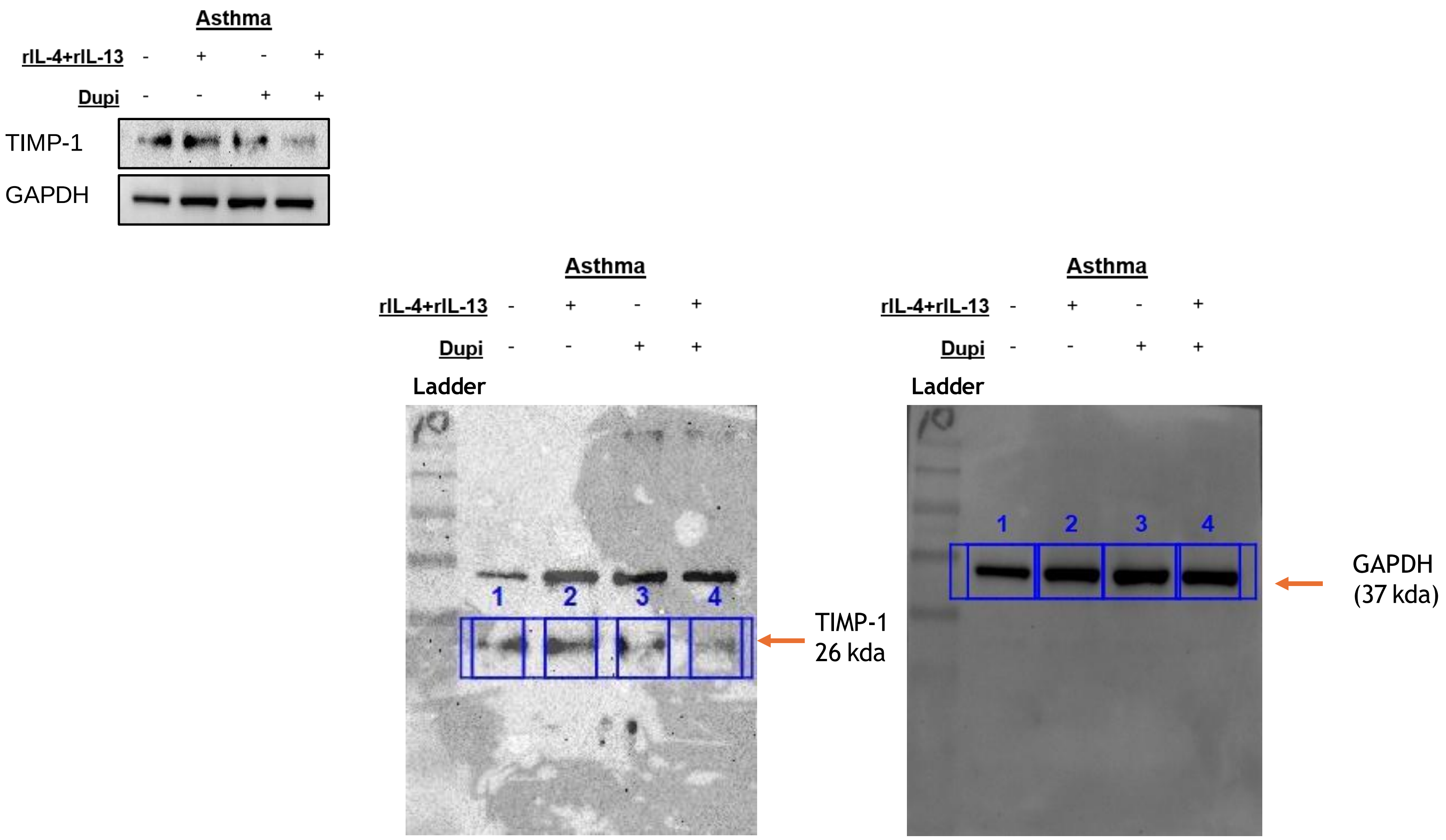

Figure 5B. Uncropped, full-length Western blot images for TIMP-1 blot upon Dupi treatment featured in the main text, with molecular weight markers and protein band labels included.
